# A Multi-Scale Approach for Phase Field Modeling of Ultra-Hard Ceramic Composites

**DOI:** 10.3390/ma14061408

**Published:** 2021-03-14

**Authors:** J. D. Clayton, M. Guziewski, J. P. Ligda, R. B. Leavy, J. Knap

**Affiliations:** DEVCOM Army Research Laboratory, Aberdeen Proving Ground, Adelphi, MD 21005, USA; matthew.c.guziewski.civ@mail.mil (M.G.); jonathan.p.ligda.civ@mail.mil (J.P.L.); richard.b.leavy.civ@mail.mil (R.B.L.); jaroslaw.knap.civ@mail.mil (J.K.)

**Keywords:** phase field, molecular dynamics, ceramics, silicon carbide, diamond, graphite

## Abstract

Diamond-silicon carbide (SiC) polycrystalline composite blends are studied using a computational approach combining molecular dynamics (MD) simulations for obtaining grain boundary (GB) fracture properties and phase field mechanics for capturing polycrystalline deformation and failure. An authentic microstructure, reconstructed from experimental lattice diffraction data with locally refined discretization in GB regions, is used to probe effects of local heterogeneities on material response in phase field simulations. The nominal microstructure consists of larger diamond and SiC (cubic polytype) grains, a matrix of smaller diamond grains and nanocrystalline SiC, and GB layers encasing the larger grains. These layers may consist of nanocrystalline SiC, diamond, or graphite, where volume fractions of each phase are varied within physically reasonable limits in parametric studies. Distributions of fracture energies from MD tension simulations are used in the phase field energy functional for SiC-SiC and SiC-diamond interfaces, where grain boundary geometries are obtained from statistical analysis of lattice orientation data on the real microstructure. An elastic homogenization method is used to account for distributions of second-phase graphitic inclusions as well as initial voids too small to be resolved individually in the continuum field discretization. In phase field simulations, SiC single crystals may twin, and all phases may fracture. The results of MD calculations show mean strengths of diamond-SiC interfaces are much lower than those of SiC-SiC GBs. In phase field simulations, effects on peak aggregate stress and ductility from different GB fracture energy realizations with the same mean fracture energy and from different random microstructure orientations are modest. Results of phase field simulations show unconfined compressive strength is compromised by diamond-SiC GBs, graphitic layers, graphitic inclusions, and initial porosity. Explored ranges of porosity and graphite fraction are informed by physical observations and constrained by accuracy limits of elastic homogenization. Modest reductions in strength and energy absorption are witnessed for microstructures with 4% porosity or 4% graphite distributed uniformly among intergranular matrix regions. Further reductions are much more severe when porosity is increased to 8% relative to when graphite is increased to 8%.

## 1. Introduction

Multi-scale computational models for nonlinear or time-dependent mechanics problems can usually be classified as concurrent or sequential. In concurrent approaches, solutions to boundary value problems at fine and coarse scales are obtained near-simultaneously, where transient feedback from the solution at the fine scale informs the solution at the coarse scale, and vice versa, as a simulation proceeds. In contrast, and simpler by construction, sequential approaches address the system’s response at different length and/or time scales consecutively, where most often, the fine-scale solution is used to provide properties or conditions invoked subsequently to establish or solve the coarse-scale problem. References encompassing both classes of approaches include [1,2,3]. A sequential multi-scale approach is used herein for modeling fracture, whereby failure properties of grain boundaries (GBs) are obtained at the fine scale via molecular dynamics (MD) simulations. These properties, namely distributions of fracture surface energies for different boundary compositions and orientations, then enter a coarse-scale representation of fracture of polycrystalline aggregates in the context of a continuum phase field model [4]. Prior works that have invoked atomistic simulations (e.g, MD or density functional theory (DFT)) to inform phase field models, via prescription of properties or functional forms of phase field energy potentials, include [5,6,7,8,9].

The aim of the present paper is an enhanced understanding of deformation and failure mechanisms in an ultra-hard dual-phase ceramic composite. The material consists of diamond and silicon carbide (SiC) crystals with complex spatial and size distributions of grains. Representative microstructures and processing routes for this material system can be found in [10,11,12,13,14,15]. In the material studied herein, disparities exist in typical grain sizes among the primary (diamond) and secondary (SiC) phases, with diamond grains tending to be at least an order of magnitude larger, with mean sizes in tens of microns. Furthermore, nanocrystalline SiC encases larger grains, and defects such as pores and graphitic inclusions may be present  [14,15]. When graphitic layers at diamond-SiC interfaces are abundant, indentation hardness is compromised [16]. Reduced stiffness, hardness, and/or fracture toughness due to graphite have also been reported for nanocrystalline diamond [17,18] and SiC [19]. Somewhat contrary to other observations, fracture toughness measured by an indentation technique was thought to increase from graphitic GB layers due to plasticity, phase transformations, and/or GB sliding mechanisms [19].

Both crystalline phases are cubic, where the most common polytype of diamond exists in concert with the β phase of SiC, which is prone to deformation twinning [20]. Although diamond is the stiffest and hardest natural material, it is brittle and prone to cleavage failure [21,22]. The SiC phase is intended to increase toughness and ductility, through a variety of crack mitigation mechanisms that have been sought in diamond-based materials via microstructure alterations [22]. During material production, the SiC phase can infiltrate GB regions, producing lower overall porosity, graphite content, and fewer debonded diamond-diamond particle contacts than would be present in polycrystalline diamond alone [11,14,16].

The theoretical phase field component of this paper most closely follows recent work in [23]. In that work, a methodology for approximating effects of graphitic inclusions and pores too small to resolve discretely in the phase field continuum was introduced, via assignment of special properties in affected regions. An idealized synthetic microstructure was studied, not directly culled from microscopy or diffraction data. Fracture energies were limited to cleavage on lowest-energy planes in each crystal phase, since sufficient information on GB energies was not available for the diamond-SiC material system. Even more limited in scope were initial studies of diamond-SiC blends in [24,25]. In all cases [23,24,25], idealized 3D polycrystalline microstructures were represented from Voronoi polyhedra, and lattice misorientation effects on GB properties were ignored. A standard variational phase field theory was used in [24], with theoretical origins in [26,27]. In [25], a geometrically enriched theory was used, with origins in  [28,29,30]).

The current work prominently extends these prior studies. New features addressed herein, with all new simulation results, include the following:

- Realistically rendered microstructures (i.e., meshes) constructed from diffraction data on real diamond-SiC samples;
- Heterogeneous distributions of GB fracture properties obtained from molecular dynamics (MD) simulations, with probed orientation distributions based on electron backscatter diffraction (EBSD) characterization;
- Superposition of GB fracture energy distributions obtained from MD with effects of defects, including subscale graphitic inclusions and initial porosity, from elastic homogenization;
- Systematic variations of phase content and scale-dependent properties (e.g., bulk microcrystals versus nanocrystalline matrix).

Results then inform structure-property-performance metrics, with an emphasis on ultimate strength and ductility of polycrystalline aggregates. Knowledge can then suggest those failure-resistant microstructures to be targeted for synthesis in future processing routes.

The outline of the remainder of this paper is as follows. Phase field representations for bulk crystals (i.e., diamond and SiC) are described in Section 2. Generation, from experimental data, of physically realistic microstructures is discussed in Section 3, along with material properties. Each subsequent section builds on the former, whereby additional physics are incorporated sequentially, leading to a more comprehensive, albeit more complex, model at each stage. Results of phase field simulations describing effects of bulk phase volume fractions and lattice orientations, with uniform fracture energies among grains of a given type or GB composition, are given in Section 4. Molecular dynamics simulations of GBs in β-SiC and diamond-SiC bicrystals that provide surface energy distributions entering the phase field fracture model are discussed in Section 5. Results of phase field simulations describing effects of heterogeneous GB fracture energy distributions are presented in Section 6. A homogenization approach to account for porosity and tertiary phases in matrix material is reviewed in Section 7. Results on effects of subscale porosity and graphitic inclusions, superposed on heterogeneous GB energies, follow in Section 8. Lastly, conclusions are given in Section 9. Vectors and higher-order tensors are written in bold-italic font, scalars in italic. Summation is implied over repeated indices in a fixed Cartesian frame when index notation is used.

## 2. Phase Field Theory: Bulk Grains and Matrix Materials

A phase field approach to modeling elasticity, deformation twinning, and fracture is invoked. The theory for bulk crystalline and matrix phases is the same as that presented in [23], which in turn has foundations in prior works that consider simultaneous twinning and fracture [24,30,31]. Prior to these, distinct models were formulated that separately considered deformation twinning [26,32,33] or fracture [27,34]. All such references, as well as the present work, use a variational approach based on global energy minimization. This approach corresponds to static equilibrium with respect to stress fields and conjugate forces to order parameters; hence, propagation speeds of crack fronts and twin boundaries are not quantitatively reproduced. Phase field formulations fully accounting for inertia and time-dependent kinetics are discussed elsewhere for brittle fracture [35,36] and deformation twinning [37,38]. Elastic strain energy density is expressed in terms of a linearized strain tensor, a representation assumed sufficient for problems of brittle fracture addressed herein, to be contemplated further upon inspection of numerical results.

### 2.1. Order Parameters

The reference location of a material particle is X, with Cartesian components $X_{K}$. The set (ξ,η) is referred to as the “internal state” of the material, where ξ=ξ(X) and η=η(X) are order parameter fields. One scalar order parameter, denoted by ξ∈[0,1], accounts for fracture:

(1) $$\begin{array}{cc} \xi (X) & =0\;\forall \;X\in \;\mathrm{undamaged}\;\mathrm{material},\\ \xi (X) & \in (0,1)\;\forall \;X\in \mathrm{partially}\;\mathrm{degraded}\;\mathrm{material},\\ \xi (X) & =1\;\forall \;X\;\mathrm{fully}\;\mathrm{failed}\;\mathrm{material}. \end{array}$$

A second scalar order parameter η∈[0,1] monitors deformation twinning:

(2) $$\begin{array}{cc} \eta (X) & =0\;\forall \;X\in \;\mathrm{parent}\;\mathrm{elastic}\;\mathrm{crystal},\\ \eta (X) & \in (0,1)\;\forall \;X\in \mathrm{twin}\;\mathrm{boundary}\;\mathrm{zone},\\ \eta (X) & =1\;\forall \;X\in \mathrm{twinned}\;\mathrm{crystal}\;\mathrm{state}. \end{array}$$

### 2.2. Kinematics

The vector field u=u(X) denotes displacement of material particles. The covariant derivative with respect to X is ∇(·), where this reduces to a partial coordinate derivative in Cartesian coordinates. The displacement gradient is decomposed into elastic distortion $\beta^{E}\left(X\right)$ and a state-dependent part $\beta^{D}$ as [30,39]

(3) $$\nabla u\left(X\right)=\beta^{E}\left(X\right)+\beta^{D}\left[\xi \left(X\right),\eta \left(X\right)\right].$$

The latter (state-dependent) term evolves in conjunction with the history of microstructure fields (ξ,η). Mechanisms of fracture and twinning provide the following summed contributions:

(4) $$\beta^{D}\left(\xi ,\eta \right)=x_{\xi }\phi_{\xi }\left(\xi \right)1+\gamma_{0}\phi_{\eta }\left(\eta \right)S⊗M.$$

Bulking is measured by the first term on the right side of (4) when $x_{\xi }\phi_{\xi }>0$, whereby residual deformation from growth of damage mechanisms—notably opening of voids or cracks—is idealized as isotropic [40,41]. The unit tensor is 1. Scalar $x_{\xi }$ is positive for expansion, consistent with bulking in rock materials [42] as well as ceramics [43]. The “2-3-4” polynomial interpolation function $\phi_{\xi }:\left[0,1\right]\to \left[0,1\right]$ obeys $\phi_{\xi }^{\prime }\left(0\right)=\phi_{\xi }^{\prime }\left(1\right)=0$, with α∈[0,6] a material constant [26,44,45]:

(5) $$\phi_{\xi }\left(\xi \right)=\alpha \xi^{2}+2\left(2-\alpha \right)\xi^{3}+\left(\alpha -3\right)\xi^{4}.$$

The maximum induced residual free volume increase is $3x_{\xi }$, occurring when ξ=1.

The second term on the right side of (4) represents shearing from deformation twins, where $\gamma_{0}$ is the simple shear induced from fully forming a twin. The habit plane for twinning is oriented by unit vector M. The twinning direction, which often correlates with the Burgers vectors of twinning partial dislocations, is oriented by the unit vector S. The isochoric simple shear for twinning [46] is the product $\gamma_{0}\phi_{\eta }$, where the interpolation function is of the same form as (5) [26,32,33]:

(6) $$\phi_{\eta }\left(\eta \right)=\chi \eta^{2}+2\left(2-\chi \right)\eta^{3}+\left(\chi -3\right)\eta^{4},$$

with χ∈[0,6]. The maximum twinning shear is $\gamma_{0}$, a material property that depends on crystal structure.

Denote the total strain tensor by ϵ. Denote the elastic strain tensor by $\epsilon^{E}$. With ${\left(\cdot \right)}^{T}$ the transpose, these symmetric tensors are

(7) $$\epsilon =\frac{1}{2}\left[\nabla u+{\left(\nabla u\right)}^{T}\right],\;\epsilon^{E}=\frac{1}{2}\left[\beta^{E}+{\left(\beta^{E}\right)}^{T}\right].$$

One argument of elastic strain energy introduced in Section 2.3 is $\epsilon^{E}=\epsilon^{E}\left(\nabla u,\xi ,\eta \right)$.

Within a single crystal region of sufficient size, numerous planes and directions are possible depending on mechanisms and microstructure. The present approach, like that in [23,24], adopts a single dominant system for each mechanism within a domain in a given single crystal so that large-scale 3D finite element calculations remain tractable. As considered in [26,47,48], distinct order parameters for each set of plane and direction could be introduced at every material point X, but with drastically increased computational expense. Anisotropic transgranular fractures were likewise monitored with a single order parameter for strength degradation in other works [23,49], wherein effects of the degree of fracture anisotropy were assessed quantitatively. Generalizations of the present approach have been devised elsewhere to model crystal-crystal and crystal-glass phase transformations [24,25,45,50].

### 2.3. Thermodynamics and Balance Laws

The total energy functional for an initially homogeneous or heterogeneous body is

(8) $$\Psi \left[u,\eta ,\xi ,X\right]=\int_{\Omega }\left[W\left(\nabla u,\eta ,\xi ,X\right)+f\left(\eta ,\xi ,\nabla \eta ,\nabla \xi ,X\right)\right]d\Omega .$$

The body occupies initial volume Ω with boundary ∂Ω and unit outward normal vector n. Properties vary with X, for example among crystals and phases in a polycrystal. Henceforth, X is dropped from lists of arguments in many functions for brevity, whereby it is understood that initial properties are homogeneous within each sub-volume corresponding to a single crystalline region [31].

For both anisotropic and isotropic materials, elastic strain energy density per unit volume *W* is of the general quadratic form

(9) $$W=W\left[\epsilon^{E}\left(\nabla u,\eta ,\xi \right),\eta ,\xi \right]=\frac{1}{2}\epsilon^{E}:C\left(\eta ,\xi ,\nabla \cdot u\right):\epsilon^{E}.$$

Denoted by C is the tensor of elastic moduli, which can depend on order parameters. Phase energy is captured by scalar function *f*. It accounts for internal state fields and their material gradients, and is partitioned as [31]

(10) $$f\left(\eta ,\xi ,\nabla \eta ,\nabla \xi \right)=f_{0}\left(\eta ,\xi \right)+g_{0}\left(\xi \right)+f_{1}\left(\xi ,\nabla \eta \right)+g_{1}\left(\nabla \xi \right).$$

Gradient-dependent terms in (10) are specified as follows, with $\kappa_{0}$ and $\omega_{0}$ constants linked to surface energies in (21):

(11) $$\begin{array}{cc} f_{1}\left(\xi ,\nabla \eta \right) & =\kappa \left(\xi \right):\left(\nabla \eta ⊗\nabla \eta \right),\;\kappa \left(\xi \right)=\kappa_{0}\hat{\iota }\left(\xi \right)1,\\ g_{1}\left(\nabla \xi \right) & =\omega :\left(\nabla \xi ⊗\nabla \xi \right),\;\omega =\omega_{0}\left[1+\hat{\beta }\left(1-N⊗N\right)\right]. \end{array}$$

The coupling degradation function $\hat{\iota }:\left[0,1\right]\to \left[0,1\right]$ introduced in [27,31] obeys $\hat{\iota }\left(0\right)=1$ and $\hat{\iota }\left(1\right)=\zeta$ and is written

(12) $$\hat{\iota }\left(\xi \right)=\zeta +\left(1-\zeta \right){\left(1-\xi \right)}^{2}.$$

Denoted by ζ is a scalar constant subject to 0<ζ≪1 that ensures a non-null residual stiffness as ξ→1. The material parameter $\hat{\beta }$ quantifies the strength of anisotropy, which for single crystals can be associated with the scarcity of multiple families of cleavage planes [23]. Setting $\hat{\beta }\gg 1$ causes anisotropic fractures to localize along cleavage planes oriented by unit normal N [34,48,49]. When $\hat{\beta }=0$, fracture energy is isotropic, the most common assumption in phase field models [51].

Governing equations for static equilibrium are obtained following usual energy methods invoking the divergence theorem and integration by parts. The first variation of total energy functional Ψ of (8) with X fixed enters the following principle:

(13) $$\delta \Psi =\oint_{S}\left(t\cdot \delta u+r\delta \eta +s\delta \xi \right)dS.$$

That part of ∂Ω over which natural boundary conditions are prescribed is S⊆∂Ω. Assuming field variables are presumed sufficiently smooth, Euler–Lagrange equations at a material point X∈Ω in (14) and (15), and natural boundary conditions on *S* in (16) are obtained as [26,27,31]

(14) $$\nabla \cdot \frac{\partial W}{\partial \nabla u}{|}_{\eta ,\xi }=\nabla \cdot P=0;$$

(15) $$\frac{\partial f}{\partial \eta }-2\nabla \cdot \left(\kappa \nabla \eta \right)+\frac{\partial W}{\partial \eta }{|}_{\nabla u,\xi }=0,\;\frac{\partial f}{\partial \xi }-2\nabla \cdot \left(\omega \nabla \xi \right)+\frac{\partial W}{\partial \xi }{|}_{\nabla u,\eta }=0;$$

(16) t=P·n,r=2κ:(∇η⊗n),s=2ω:(∇ξ⊗n).

Stress tensor P is symmetric and divergence-free in equilibrium in the absence of body forces and inertia. Mechanical traction is the vector t. The conjugate surface force to the twinning order parameter η is *r*, and the conjugate surface force to the fracture order parameter ξ is *s*.

### 2.4. Energy Density, Governing Equations, and Numerical Techniques

Although anisotropic and nonlinear elastic models have been invoked elsewhere in phase field simulations [31,32,33,34], computational cost is high. Here, as justified in [23,24,25], isotropic linear elasticity is chosen for simplicity and efficiency. Strain energy density per unit volume of (9) becomes

(17) $$W=\frac{1}{2}\lambda {\left(\mathrm{tr}\epsilon^{E}\right)}^{2}+\mu \epsilon^{E}:\epsilon^{E}.$$

Lamé coefficients λ and μ degrade commensurately with increases in ξ, to account for damage softening. The sign of elastic volume change is used to account for differences in softening of the bulk modulus in tension and compression:

(18) $$\mu \left(\xi \right)=\mu_{0}\left[\zeta +\left(1-\zeta \right){\left(1-\xi \right)}^{2}\right],\;\lambda \left(\xi ,\mathrm{tr}\epsilon^{E}\right)=k\left(\xi ,\mathrm{tr}\epsilon^{E}\right)-\frac{2}{3}\mu \left(\xi \right);$$

(19) $$k\left(\xi ,\mathrm{tr}\epsilon^{E}\right)=\left(\lambda_{0}+\frac{2}{3}\mu_{0}\right)\left\{\left[\zeta +\left(1-\zeta \right){\left(1-\xi \right)}^{2}\right]\langle \mathrm{tr}\epsilon^{E}\rangle +{\langle -\mathrm{tr}\epsilon^{E}\rangle }^{*}\right\}.$$

As introduced in [27], the operator 〈x〉=1∀x>0, 〈x〉=0∀x≤0, ${\langle x\rangle }^{*}=1\forall x\geq 0$, and ${\langle x\rangle }^{*}=0\forall x<0$.

Tangent bulk modulus *k* tends to degrade with fracture under tensile pressure but not under compressive pressure. This prescribes inviscid fluid-like behavior for fully degraded zones, i.e., non-zero pressure in compression but vanishing shear strength as ξ→0. The tangent shear modulus changes equivalently with respect to ξ in both tension and compression.

The two scalar phase energy functions $\left(f_{0},g_{0}\right)$ that depend on order parameters, but not their gradients, entering (10) are

(20) $$\begin{array}{c} f_{0}\left(\eta ,\xi \right)=A\eta^{2}{\left(\eta_{0}-\eta \right)}^{2}\cdot H\left(\eta_{0}-\eta \right)\cdot \hat{\iota }\left(\xi \right),\;g_{0}\left(\xi \right)=B\xi^{2}. \end{array}$$

The first term in $f_{0}$ is a double-well potential with minima at $\eta =(0,\eta_{0})$, a local maximum at $\eta =\eta_{0}/2$, and a cut-off demarcated by $\eta_{0}>0$ [24,25]. The Heaviside step function is H(·). Traditionally, $\eta_{0}=1$ is used to model isolated twinning [26,32], the simplest physically realistic assumption used herein as in [23]. Other values may be applicable to account for solid–solid phase changes, which may occur in conjunction with twinning in certain materials [9,24,25,30]. Function $g_{0}$ is the standard quadratic form used in phase field modeling of fracture [27,51,52].

Denote by Γ the twin boundary energy per unit reference area, often closely related to intrinsic stacking fault energy associated with twinning partial dislocations [46,53,54]. Let Υ denote the fracture surface energy per unit reference two-sided area. Denote two potentially different regularization lengths of phase field theory by $l_{\eta }$ and $l_{\xi }$. From (11) and (20), extension of derivations in [26,31] produces the following definitions of *A* and *B* (energies per unit volume) and $\kappa_{0}$ and $\omega_{0}$ (energies per unit length) [24]:

(21) $$A=12\frac{\Gamma }{\eta_{0}^{4}l_{\eta }},\;\kappa_{0}=\frac{3}{4}\frac{\Gamma l_{\eta }}{\eta_{0}^{2}};\;B=\frac{\Upsilon }{l_{\xi }},\;\omega_{0}=\Upsilon l_{\xi }.$$

The finite element (FE) method is used to solve 3D boundary value problems in an incremental fashion, where the load history is partitioned into small steps to monitor the path-dependent response in the presence of twinning and fractures. Boundary conditions are updated during each load step. Algorithms that seek equilibrium apply conjugate gradient energy minimization: candidate fields u(X),η(X),ξ(X) are obtained that minimize Ψ subject to boundary constraints and thus fulfill (13). To enforce irreversibility of crack extension, constraints on evolution of ξ once exceeding a threshold value $\xi_{T}$ are imposed. Similarly, a threshold value $\eta_{T}$ is imposed to render twinning irreversible. Details on numerical procedures are available in [24,26,30,31,55]. Specifically, the FE model is implemented in a custom, massively parallel C++ MPI (message passing interface) framework [55].

Specific governing equations to be solved numerically are obtained from substitution of constitutive functions in (10), (11), (17), and (20) into Euler–Lagrange Equations (14) and (15). The partial derivatives listed below, which correspond to the constitutive law for stress and various contributions to phase-dependent thermodynamic conjugate forces, enter the procedure:

(22) $$P=\frac{\partial W}{\partial \nabla u}{|}_{\eta ,\xi }=\lambda \left(\mathrm{tr}\epsilon^{E}\right)1+2\mu \epsilon^{E}+\frac{1}{2}\lambda {\left(\mathrm{tr}\epsilon^{E}\right)}^{2}\frac{\partial k}{\partial (\mathrm{tr}\epsilon^{E})}1,$$

(23) $$\frac{\partial f}{\partial \eta }=\left\{\frac{d}{d\eta }\left[A\eta^{2}{\left(\eta_{0}-\eta \right)}^{2}H\left(\eta_{0}-\eta \right)\right]\right\}\left[\zeta +\left(1-\zeta \right){\left(1-\xi \right)}^{2}\right],$$

(24) $$\frac{\partial W}{\partial \eta }{|}_{\nabla u,\xi }=\tau =\mu \phi_{0}^{\prime }\gamma_{0}\left[\phi_{0}\gamma_{0}-\nabla u:\left(S⊗M+M⊗S\right)\right]\approx -\phi_{0}^{\prime }\gamma_{0}P:S⊗M,$$

(25) $$\frac{\partial f}{\partial \nabla \eta }=2\kappa \nabla \eta ,\;\frac{\partial f}{\partial \nabla \xi }=2\omega \nabla \xi ,$$

(26) $$\frac{\partial f}{\partial \xi }=2B\xi -2\left[\left(1-\zeta \right)\left(1-\xi \right)\right][\kappa_{0}{\left|\nabla \eta \right|}^{2}+A\eta^{2}{\left(\eta_{0}-\eta \right)}^{2}H\left(\eta_{0}-\eta \right)],$$

(27) $$\frac{\partial W}{\partial \xi }{|}_{\nabla u,\eta }=\varsigma =\frac{1}{2}\left(\frac{\partial k}{\partial \xi }-\frac{2}{3}\frac{\partial \mu }{\partial \xi }\right){\left(\mathrm{tr}\epsilon^{E}\right)}^{2}+\frac{\partial \mu }{\partial \xi }\epsilon^{E}:\epsilon^{E}-3k\left(\mathrm{tr}\epsilon^{E}\right)\phi_{\xi }^{\prime }x_{\xi },$$

(28) $$\frac{\partial k}{\partial \xi }=-2\left(\lambda_{0}+\frac{2}{3}\mu_{0}\right)\left\{\left[\left(1-\zeta \right)\left(1-\xi \right)\right]\langle \mathrm{tr}\epsilon^{E}\rangle \right\},\;\frac{\partial \mu }{\partial \xi }=-2\mu_{0}\left(1-\zeta \right)\left(1-\xi \right).$$

The elastic driving force for deformation twinning is ${\tau =\left(\partial W/\partial \eta \right)|}_{\nabla u,\xi }$, closely related to the resolved shear stress acting on habit plane M in direction S. The elastic driving force for cavitation is the rightmost term in (27) for ς, which is $\approx 3\phi_{\xi }^{\prime }x_{\xi }p$ with $p=-\frac{1}{3}\mathrm{tr}P$ the Cauchy pressure.

## 3. Materials and Microstructures

The material system of study consists of bulk diamond and SiC single crystals embedded in a matrix of smaller SiC grains (micro- and nano-crystals), with layers surrounding larger crystals that may contain nanocrystalline graphite, nanocrystalline SiC, or much smaller diamond grains. Graphite and pores may also be embedded in the matrix material, as addressed later in Section 7.

### 3.1. Properties for Diamond, β-SiC, and Graphite

Properties entering the phase field theory for each crystalline constituent are given in Table 1. Individual values are justified, with source references, for each material in Section 3.1.1, Section 3.1.2 and Section 3.1.3. Nominal properties correspond to larger, individually resolved crystals in the case of diamond and SiC, where cleavage fracture is planar and anisotropic. Twinning is permissible in such cases in the SiC phase. Furthermore, dilatation is assumed negligible ($x_{\xi }=0$) whereby fracture surfaces are considered smooth within a single crystal region. Each grain is idealized as elastically isotropic, with the same elastic constants. Graphitic regions are polycrystalline and also treated as isotropic. Fracture energies of cleavage planes of bulk crystals are identical among crystals of a given phase (i.e., diamond or SiC), but fracture behaviors differ among single crystals due to different orientations of cleavage planes in randomly oriented grains.

When a material element is assumed to consist of numerous randomly oriented nanocrystals, its inelastic response is assume isotropic. Thus, twinning is not resolved in the homogenized nanocrystalline matrix as no preferred habit plane and twinning direction exist for an isotropic assembly. Dilatation from bulking is non-zero since fractures are more tortuous, spanning subscale planes and GBs of orientations not perfectly aligned. The maximum local value used, $x_{\xi }=0.04$, is comparable to that justified in prior works on boron carbide [30,39] and SiC [23]. Finite dilatation is consistent with observed behaviors of many polycrystalline rocks and minerals that expand inelastically under uniaxial or triaxial compression [42].

#### 3.1.1. Diamond

Cubic diamond (a zinc blende structure) is a member of space group Fd$\bar{3}$m with lattice parameter 0.357 nm and mass density 3.52 g/cc. Diamond is modeled as elastic-brittle since plastic slip, twinning, and phase changes do not occur for stress states and temperatures presently studied. Parameters Γ, $\gamma_{0}$, and χ are not needed. Anisotropic single crystal constants $C_{11}$, $C_{12}$, and $C_{44}$ are used to calculate the unique bulk modulus $k_{0}$ and the Hill shear modulus $\mu_{0}$, where the latter is the mean of Voigt and Reuss bounds [56]:

(29) $$k_{0}=\frac{1}{3}\left(C_{11}+2C_{12}\right),\;\mu_{0}=\frac{1}{10}\left(C_{11}-C_{12}+3C_{44}\right)+\frac{5}{2}{\left[4/\left(C_{11}-C_{12}\right)+3/C_{44}\right]}^{-1}.$$

The cubic anisotropy of single crystalline diamond is very low (i.e., $C_{11}-C_{22}\approx 2C_{44}$ [21,23]), so the isotropic elasticity assumption is reasonable. Fracture energy corresponds to cleavage on {111} planes with unit normal N, those of lowest surface energy [21]. Anisotropy of fracture energy follows from the value of $\hat{\beta }$ listed in Table 1. Values of $\hat{\beta }$ and α are presumed universal among ceramics of present interest, and of those studied in prior works on twinning and fracture [27,31,32,33,34]. The minimum ratio of regularization length $l=l_{\eta }=l_{\xi }$ to bulk diamond grain size $l_{G}$ in Table 1 is dictated by mesh resolution. Gradients of order parameter fields must captured adequately by the numerical discretization. As shown later, large diamond bulk crystals are discretized individually into $\approx 10^{4}$ full-integration hexahedral finite elements.

From anisotropic single crystal elastic constants [21], the anisotropy of diamond is very low, with the Zener ratio $2C_{44}/\left(C_{11}-C_{12}\right)=1.13$. Thus, the use of isotropic elasticity for diamond is reasonable.

#### 3.1.2. Silicon Carbide

Silicon carbide demonstrates a number of common polytypes, including those of cubic, hexagonal, and rhombohedral structures. The former dominates microstructures of present interest: the 3C polytype (β phase) inhabits space group F$\bar{4}$3m (zinc blende) with lattice parameter 0.436 nm and mass density 3.21 g/cc. Crystals may undergo fracture and deformation twinning, respectively, quantified by order parameter fields ξ(X) and η(X). Isotropic elastic constants are obtained from anisotropic single crystal constants for cubic SiC given by [57] inserted into (29). Elastic anisotropy is moderate for SiC, but as justified in [23], prior research [34] showed that elastic anisotropy has a small effect on phase field fracture predictions relative to cleavage energy anisotropy. Cleavage fracture planes are {110}, giving N. Fracture energy Υ for {110} planes is obtained from [58]. Twinning [20] occurs on $\langle 11\bar{2}\rangle \left\{111\right\}$, giving S and M, with magnitude $\gamma_{0}=\sqrt{2}/2$ [46,59]. Intrinsic stacking fault energy Γ is associated with a twin boundary energy per unit (one-sided) area  [26,46,53]. The experimental value [60] is notably low, leading to profuse stacking faults in addition to deformation twins [14]. Interpolation constant χ is justified in [26,32,33]. Identical values of $x_{\xi }$, α, and *l* are used for SiC and diamond, as well as graphite; no physical basis exists for a difference.

For 3C-SiC, anisotropy is moderate, with a Zener ratio of 2.06 [57]. Previous phase field simulations incorporating anisotropic elasticity and anisotropic fracture using a similar framework, but without deformation twinning [34], showed that crack propagation was relatively unaffected by anisotropic elastic properties of SiC grains, but was significantly affected by cleavage plane orientation for $\hat{\beta }\geq 10$. Thus, effects of anisotropic elasticity should be secondary to those of anisotropic fracture in the present simulations.

Computational demands of coupling anisotropic elasticity with a phase field model of anisotropic fracture and deformation twinning render the approach intractable for 3D problems of the current scope. Complexity is increased immensely when twinning is possible, since anisotropic elastic constants should be transformed in twinned regions to account for rotation or reflection of the crystal lattice across the habit plane [61]. A validated means of degrading anisotropic elastic coefficients in a directionally-dependent way due to fracture, while concurrently transforming anisotropic elastic coefficients due to twinning, remains to be established.

Hexagonal and cubic single crystal polytypes of SiC are both non-centrosymmetric semiconductors with weakly piezoelectric properties [62,63]. Electromechanical effects are deemed unimportant for brittle polycrystals with random orientations studied here [63]. Pressures incurred in numerical simulations herein are beneath the transformation threshold to a rock salt structure [64].

#### 3.1.3. Graphite

Graphite is a common crystalline form of carbon with hexagonal symmetry. Single crystals are highly anisotropic, but the present phase field representations address material elements comprised of many smaller nanocrystalline entities of random orientation, with isotropic elastic properties overall [19,23]. The Hill averaging scheme [56] corresponding to the arithmetic mean of Voigt and Reuss bounds is used to calculate effective isotropic elastic constants from anisotropic single crystal values. For hexagonal symmetry, the latter are $C_{11}$, $C_{12}$, $C_{13}$, $C_{33}$, and $C_{44}$, where $C_{66}=\frac{1}{2}\left(C_{11}-C_{12}\right)$. Values are obtained from [65]. Define $m=C_{11}+C_{12}-4C_{13}+2C_{33}$ and $c^{2}=\left(C_{11}+C_{12}\right)C_{33}-2C_{13}^{2}$. Then $k_{0}$ and $\mu_{0}$ are evaluated as [66]

(30) $$\begin{array}{cc} k_{0} & =\frac{1}{2}\left[\frac{2\left(C_{11}+C_{12}\right)+C_{33}+4C_{13}}{9}+\frac{c^{2}}{m}\right],\\ \mu_{0} & =\frac{1}{2}\left[\frac{1}{30}\left(m+12C_{44}+12C_{66}\right)+\frac{5}{2}\frac{c^{2}C_{44}C_{66}}{3k_{V}C_{44}C_{66}+c^{2}\left(C_{44}+C_{66}\right)}\right], \end{array}$$

where $9k_{V}=2\left(C_{11}+C_{12}\right)+C_{33}+4C_{13}$. Voigt and Reuss bounds are respective first and second set of terms in square braces. The value listed in Table 1 for Υ is for separation of (0001) planes [67], the weak link in the material that should dominate fracture behavior. Possible high-pressure transformations to hexagonal diamond structure [68] are outside the present scope of loading conditions. Smooth fractures on basal planes are assumed as graphite is a natural dry lubricant; hence, $x_{\xi }=0$.

### 3.2. Microstructures

The composite ceramic of study is obtained from M Cubed Technologies (Newark, DE, USA). Specimens are sectioned serially, and then exposed surfaces are characterized via electron backscatter diffraction (EBSD), enabling identification of different phases and crystal lattice orientations within each phase.

**Figure 1:**
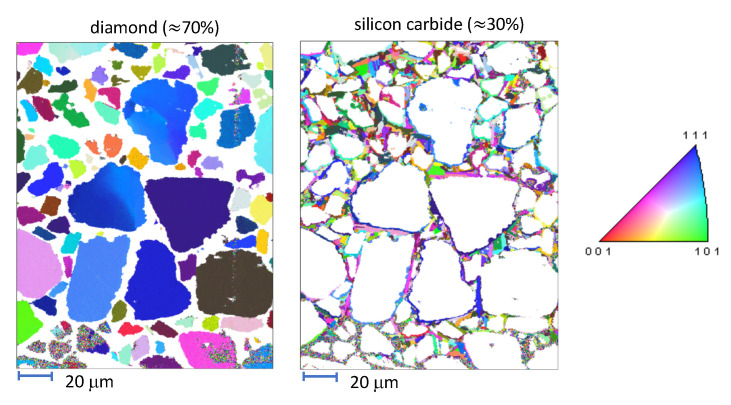
Orientation map (inverse pole figure) from EBSD scan of diamond-SiC sample: diamond lattice orientations on left, silicon carbide on right.

**Figure 2:**
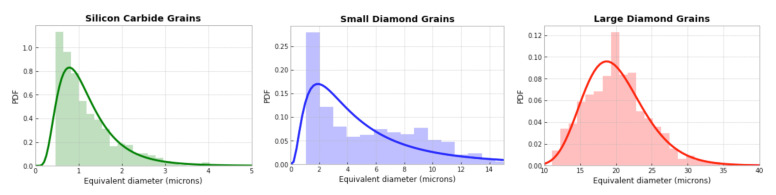
Grain size probability distribution functions (PDFs) for experimental diamond-SiC sample, with diamond split into two slightly overlapping sets. Curves are lognormal fits.

A characteristic microstructure is shown in Figure 1. This material, fully dense (no observed porosity), consists of ≈70% by volume diamond and ≈30% SiC. Impurities, including graphite, total less than 1% by volume. Larger diamond grains have average diameters around 20 µm. Smaller diamond grains have diameters ranging from 1 to 15 µm. Discrete SiC grains have diameters ranging from 0.5 to 5 µm. Grain size distributions are shown in Figure 2. The remainder of the material in the experimental samples is a SiC matrix (termed nanocrystalline, grain sizes < 0.5 µm). Lattice orientations, distinguished by color in Figure 1, are random in both phases.

The computer rendering of the microstructure is created as follows. First, statistical data is imported into the dream3d software (v6.5.138, http://dream3d.bluequartz.net (accessed on 1 October 2020)), wherein a voxel mesh is constructed and exported. The voxel mesh is then imported into cubit software (v15.6, https://cubit.sandia.gov (accessed on 1 October 2020)), wherein it is transformed to a smoothed hexahedral FE mesh. Finally, scripting steps are undertaken to assign GB layer regions surrounding larger diamond crystals, and to subdivide matrix material into manageable “grains” among which phases and material properties may vary. The FE mesh consist of approximately 1.5 M full-integration elements, with selective refinement in GB regions. Consistent with mesh densities used in prior works [23,31,34], refinement is sufficient to resolve relatively sharp order parameter gradients in interfacial zones. The total number of bulk grains among which material properties may vary is 1056, plus the GB layer that consists of distinct 170 regions, with each region encasing a larger diamond crystal.

**Figure 3:**
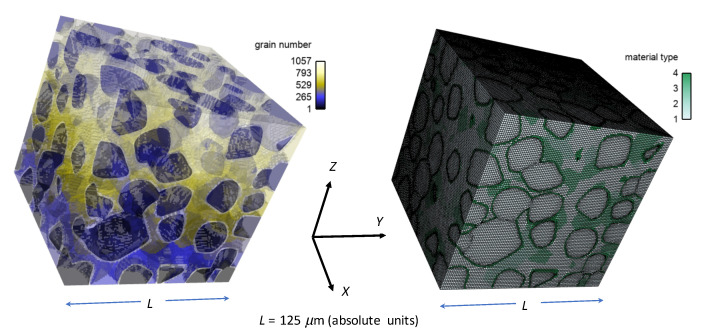
Reconstructed microstructure based on diamond-SiC characterization data. Grains numbered on left assign a unique number to each discrete grain and to each representative block of SiC matrix (refer to text of Section 3.2). All 170 GB layers are assigned the same “number” (here, 1057; properties that may vary among layers). Mesh with material types on right, where 1 = diamond (anisotropic), 2 = SiC micro-crystals (anisotropic), 3 = SiC matrix (isotropic), 4 = GB layer phase (isotropic nanocrystalline; can be SiC, diamond, or graphite).

The microstructure and FE mesh are visualized in Figure 3. The total volume fraction of diamond (70%) is reproduced exactly from the experimental microstructure, and the average equivalent diameter of the 170 large diamond grains in the FE representation, 20.5 µm, is consistent with the data of Figure 2 (far right). The 531 smaller diamond grains and 178 discrete SiC grains are of average size 10 µm, which is larger than experimental sizes in Figure 2, a noted limitation of the modeling. Smaller discrete grains of size on the order of 1 µm or less could not be resolved explicitly due to meshing constraints. Instead, in the FE model, such very small SiC grains are included in the homogeneous matrix.

Eighteen different combinations of microstructure and loading direction are listed in Table 2. Results from simulations for these cases are discussed in Section 4. These simulations are denoted by the sequence α.β.γ, where α=a corresponds to the present set. Cases α=b,c will be introduced later in Section 5 and Section 7, respectively. The qualifier “β” here runs from 1 to 6 and defines the phase content. The qualifier “γ” is x, y, or z, indicative of the corresponding uniaxial loading direction: *X*, *Y*, or *Z*, respectively, in Figure 3. Boundary conditions are defined explicitly in Section 3.3.

The baseline microstructure, most consistent with experimental characterization, is labeled a.1.γ. The total diamond fraction is 70%, with the remainder all SiC, partitioned into moderate distinct crystals (anisotropic fracture and twinning) and matrix phase (isotropic fracture, no twinning). The GB layers surrounding diamond grains nominally consist of the SiC matrix material. Other simulations systematically vary phase content to enable interrogation of structure-property-performance of virtual composite materials. Simulations a.2.γ and a.3.γ replace the GB layer material with nanocrystalline graphite or a diamond matrix, respectively. In the latter case, the total volume fraction of diamond increases to 82.6%. In simulations a.4.γ, the fraction of SiC grains is larger, with no SiC matrix except the GB layers. In simulations a.5.γ, all SiC material is taken as isotropic matrix. In simulations a.6.γ, the larger diamond grains are maintained, but all moderate-sized diamond crystals from the nominal case (β=1,…,5) are switched to SiC crystals. In this final case, the volume fraction of diamond is only 39.4%.

Lattice orientations are assigned randomly among grains within a given microstructure. These affect the constitutive model via the unit vectors S and M for twinning and N for fracture planes. The same orientation distribution is used for all β=1,…,6 with γ held fixed. However, a different orientation set is used for each of γ=x,y,z. Thus, distinctive lattice orientation effects manifest with changes in γ but not with changes in β. Summarizing, differences among simulation results at fixed α,γ are due to phase content, while differences among results at fixed α,β are due to orientation of the grain structure and crystal lattices.

The global edge length of the composite domain Ω is $L=\frac{25}{4}l_{G}$, with $l_{G}$ defined as the mean diameter of bulk diamond crystals, around 20 µm in absolute units for experimental samples such as that shown in Figure 1. In forthcoming numerical simulations, $l=0.04l_{G}=0.8$ nm where $l_{G}\to 10^{-3}l_{G}$ such that fracture processes, and overall strength, of small-scale samples are realistically resolved. This rescaling has been validated in prior work [23] by comparison of results for peak compressive strength with experiments on polycrystalline SiC [69,70,71]. The original absolute size of grains, in the 1–100 micron range, cannot be discretized using elements of nm dimensions, since the total number of elements becomes too large to enable numerical solutions in any reasonable wall-clock time. Therefore, spatial dimensions are rescaled downwards consistently to provide accurate predictions of fracture strength $P_{C}$, which scales as $P_{C}\propto \sqrt{\Upsilon /l}$ in a homogeneous body under simple tension [52] or simple shear [72]. Regularization lengths on the order of nm were used elsewhere in phase field studies of fracture and phase transformations [45,73,74], and notably in [27] where verification with analytical solutions was presented. Regularization parameter *l* enters the variational problem only through the normalization of energetic contributions in *f* of (10) to the free energy functional Ψ that depend exclusively on order parameters and their gradients. Length rescaling would have further consequences in dynamic problems involving wave propagation not addressed here. The present length transformation ($l\to 10^{-3}l$, $\Omega \to 10^{-9}\Omega$) produces an energy functional per unit volume Ψ/Ω equivalent to that which would be achieved by increasing the fracture surface energy by the inverse amount ($\Upsilon \to 10^{3}\Upsilon$). The latter, essentially choosing the fracture surface energy based on a pre-assigned fracture strength $P_{C}$ and regularization length *l*, is an analogous approach to avoid spuriously low fracture strength in phase field models when the regularization length *l* is too large due to minimum mesh size constraints [51,75].

### 3.3. Boundary Conditions

Uniaxial stress compression conditions are applied. Displacement boundary conditions on two faces of ∂Ω are enforced, and Ω is constrained to eliminate rigid body modes. One face is fixed in the loading direction, with displacement increment $\delta_{i}$ applied uniformly to the opposite face for increment *i*. Nominal axial strain, positive in compression, is

(31) $$\bar{\epsilon }=\frac{1}{L}\sum_{i}\delta_{i}.$$

Lateral surfaces are free to expand to maintain an overall 1D stress state: traction t of (16) vanishes on lateral faces. For a linear isotropic solid with constant Young’s modulus $E_{0}$, the average axial stress incurred would be $\bar{P}=E_{0}\bar{\epsilon }$. Free natural boundary conditions r=s=0 are assigned everywhere on ∂Ω [see (16)]. These permit twins and cracks to percolate unimpeded across the entire domain. Recall from Section 2 that a variational approach based on global energy minimization is used, which corresponds to static equilibrium with respect to stress fields and conjugate forces to order parameters at each load increment. The model incorporates no rate dependent effects (e.g., viscosity or inertia), and loading is quasi-static.

### 3.4. Local and Global Stress Measures and Order Parameters

Recall Ω=∫dΩ is total initial volume of the body. Average stress and twin fraction are defined, respectively, as

(32) $$\bar{P}=\frac{1}{\Omega }\int_{\Omega }P\left(X\right)\;d\Omega ,\;\bar{\eta }=\frac{1}{\Omega }\int_{\Omega }\eta \left(X\right)\;d\Omega .$$

Local pressure is $p\left(X\right)=-\frac{1}{3}P_{KK}\left(X\right)$. Local effective (Mises) stress, non-negative by construction, is $\sigma \left(X\right)={\left\{\frac{1}{2}\left[{\left(P_{11}-P_{22}\right)}^{2}+{\left(P_{22}-P_{33}\right)}^{2}+{\left(P_{33}-P_{11}\right)}^{2}+6\left(P_{12}^{2}+P_{23}^{2}+P_{31}^{2}\right)\right]\right\}}^{1/2}$, where $P_{IJ}=P_{IJ}\left(X\right)$. Average pressure and effective stress, the latter calculated using the first of (32), are, respectively,

(33) $$\begin{array}{cc} \bar{p} & =-\frac{1}{3}{\bar{P}}_{KK},\\ \bar{\sigma } & =\{\frac{1}{2}\left[{\left({\bar{P}}_{11}-{\bar{P}}_{22}\right)}^{2}+{\left({\bar{P}}_{22}-{\bar{P}}_{33}\right)}^{2}+{\left({\bar{P}}_{33}-{\bar{P}}_{11}\right)}^{2}+6\left({\bar{P}}_{12}^{2}+{\bar{P}}_{23}^{2}+{\bar{P}}_{31}^{2}\right)\right]{\}}^{1/2}. \end{array}$$

Calculations give $\bar{\sigma }$ values usually smaller than the volume average σ since positive and negative components counteract more often in the former. A cumulative energy density is defined as the path integral over the loading history of a simulation, denoted as the stress work [23]:

(34) $$\bar{w}=\int \bar{P}d\bar{\epsilon }\;\left[\mathrm{compression}\;\mathrm{loading}\;\mathrm{in}\;j-\mathrm{direction},\;\bar{P}=-{\bar{P}}_{jj}\;\left(\mathrm{no}\;\mathrm{sum}\;\mathrm{on}\;j\right)\right].$$

## 4. Phase Field Results: Bulk and Layer Composition Effects

Simulations listed in Table 2 consider different phase content among bulk grains, matrix regions, and GB layers encasing 170 diamond grains with average size of 20.5 µm, as explained in Section 3.2. The 70% diamond fraction corresponds to the experimental sample, while the 39.4% diamond fraction is an excursion to investigate the response of a material with less diamond [13]. Partitioning of SiC into 9.4% grains and 9.0% matrix is likewise consistent with the real sample, while other choices are excursions that are convenient based on mesh geometry. The 11.6% layer volume fraction is necessitated by mesh resolution, since a finer mesh would be required to resolve a much thinner layer. The default material for this layer is SiC, based on the real sample, but graphite and diamond layers are also studied to obtain further understanding, since these have been observed in other diamond-SiC materials [12,14,16]. Effects of these different compositions on local and global mechanical behaviors are revealed in what follows next. Displacement boundary conditions are imposed in terms of normal strain $\bar{\epsilon }\geq 0$, defined in (31), acting in the global *X*, *Y*, or *Z* direction.

**Figure 4:**
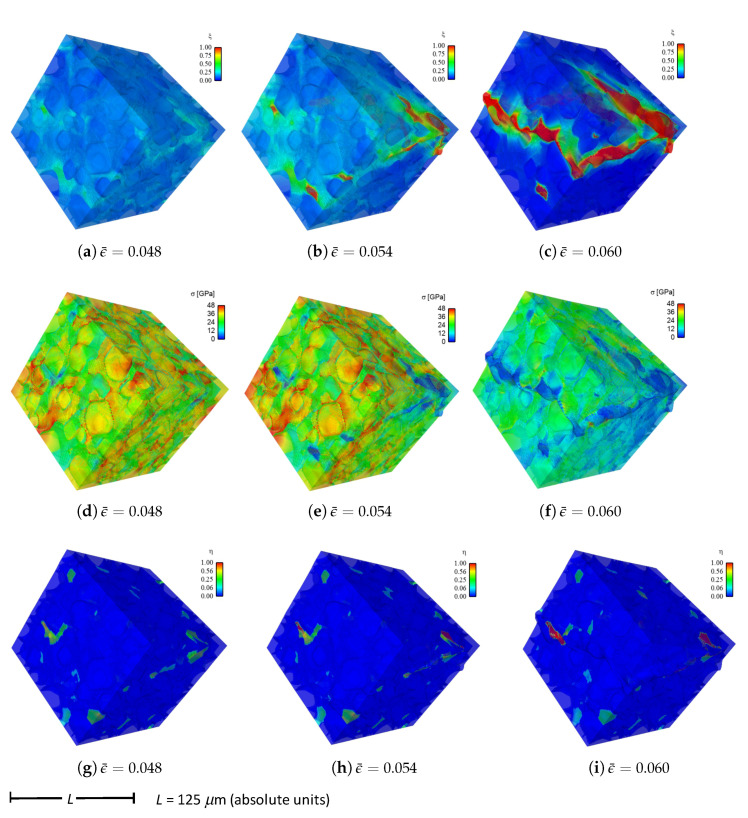
Stress and order parameter fields for simulation a.1.y (70% diamond, mixed SiC grains and matrix, SiC GB layers): (**a**) fracture order parameter ξ, $\bar{\epsilon }=0.048$, (**b**) fracture order parameter ξ, $\bar{\epsilon }=0.054$, (**c**) fracture order parameter ξ, $\bar{\epsilon }=0.060$, (**d**) effective stress σ, $\bar{\epsilon }=0.048$, (**e**) effective stress σ, $\bar{\epsilon }=0.052$, (**f**) effective stress σ, $\bar{\epsilon }=0.060$, (**g**) twinning order parameter η, $\bar{\epsilon }=0.048$, (**h**) twinning order parameter η, $\bar{\epsilon }=0.052$, (**i**) twinning order parameter η, $\bar{\epsilon }=0.060$.

Shown in Figure 4 are contours of field variables for simulation a.1.y, where compression is along the *Y*-direction of Figure 3. The microstructure, which is considered most physically representative of experimental characterization data discussed in Section 3.2, contains 70% total fraction of diamond and 30% SiC. The SiC is partitioned among discrete grains (9.4%), isotropic matrix (9.0%), and isotropic GB layers (11.6%). Snapshots at increasing strain values are given from left to right, while each horizontal set of three subfigures shows the fracture order parameter, the local effective (von Mises) stress, and the twinning order parameter. Fractures initiate in the weaker SiC matrix and along grain and phase boundaries. Fractures propagate to include cleavage across diamond crystals, leading to percolation for $\bar{\epsilon }≳0.50$. Twinning is restricted to discrete granular SIC regions, which occupy only a small fraction of the diamond-heavy composite. It arises selectively only in crystals favorably oriented with respect to Schmid factor. Stresses tend to be larger in stiffer diamond regions, and stress concentrations are evident at phase boundaries. Stresses relax at larger strains as a result of stiffness degradation commensurate with fracture, as ξ→1 within cracked regions.

**Figure 5:**
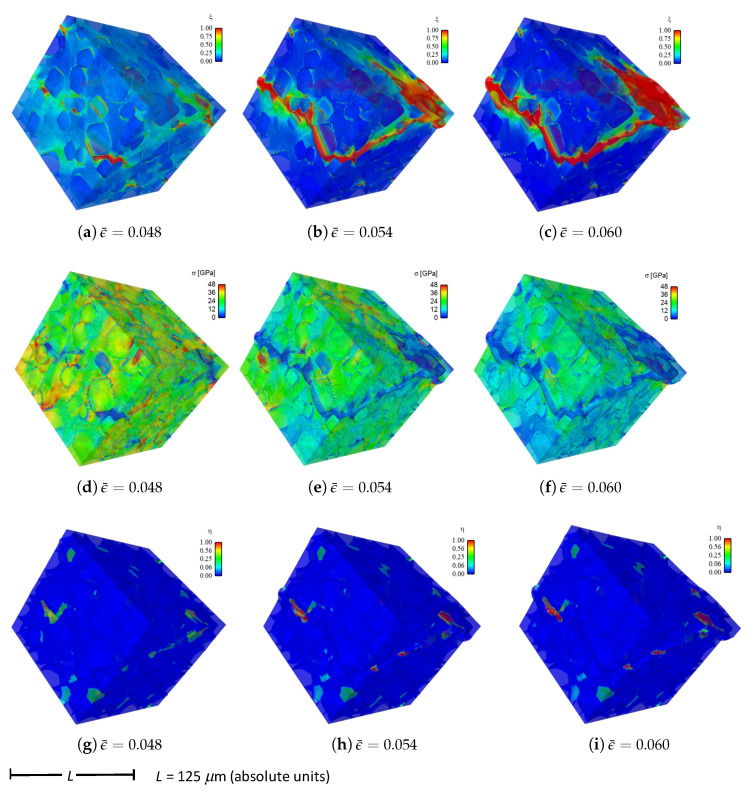
Stress and order parameter fields for simulation a.2.y (70% diamond, mixed SiC grains and matrix, graphite GB layers): (**a**) fracture order parameter ξ, $\bar{\epsilon }=0.048$, (**b**) fracture order parameter ξ, $\bar{\epsilon }=0.054$, (**c**) fracture order parameter ξ, $\bar{\epsilon }=0.060$, (**d**) effective stress σ, $\bar{\epsilon }=0.048$, (**e**) effective stress σ, $\bar{\epsilon }=0.052$, (**f**) effective stress σ, $\bar{\epsilon }=0.060$, (**g**) twinning order parameter η, $\bar{\epsilon }=0.048$, (**h**) twinning order parameter η, $\bar{\epsilon }=0.052$, (**i**) twinning order parameter η, $\bar{\epsilon }=0.060$.

Reported in Figure 5 are contours of field variables for simulation a.2.y, again compressed along the *Y*-direction of Figure 3. The microstructure contains 70% total fraction of diamond, 18.4% SiC, and 11.6% graphite. The SiC is partitioned among discrete grains (9.4%) and isotropic matrix (9.0%). Graphite comprises the GB layers (11.6%) encasing the large diamond crystals. The same lattice orientation distribution is used here and in simulation a.7.y. The only difference between the current case and that shown in Figure 4 is the presence of nanocrystalline graphite, rather than nanocrystalline SiC, at GB layers. Fractures often initiate in the graphite phase, which has notably low surface energy: 0.20 J/m${}^{2}$ for graphite versus 2.33 J/m${}^{2}$ for SiC in Table 1. Fractures later propagate to matrix material and transgranular modes across diamond crystals, leading to percolation for $\bar{\epsilon }≳0.49$. Twinning behavior in Figure 5 is slightly more prevalent than in Figure 4, though differences among the two cases are small. Stresses are often larger in stiffer diamond regions, and are often low in graphitic regions due to softening commensurate with fracture in the much weaker graphite phase. Relative to the baseline simulation results of Figure 4, those in Figure 5 show earlier and more severe fractures (ξ) as well as lower overall effective stresses σ at the same applied strain $\bar{\epsilon }$.

Fluid-like behavior (ξ→1) is observed for the pulverized SiC-graphite material in the upper right of parts (c), (f), and (i) of Figure 5. As discussed in [23], accuracy of a linear elastic theory is thought acceptable for bulk grains where strains remain modest. Linear elasticity is presumably less accurate for highly distorted regions in damaged material at later load steps in the simulation history, e.g., the upper right corner of the domain in Figure 5c. No known elastic potential, linear or nonlinear, is thought to be fully validated for such regions of very high crack density, wherein strain softening is severe.

**Figure 6:**
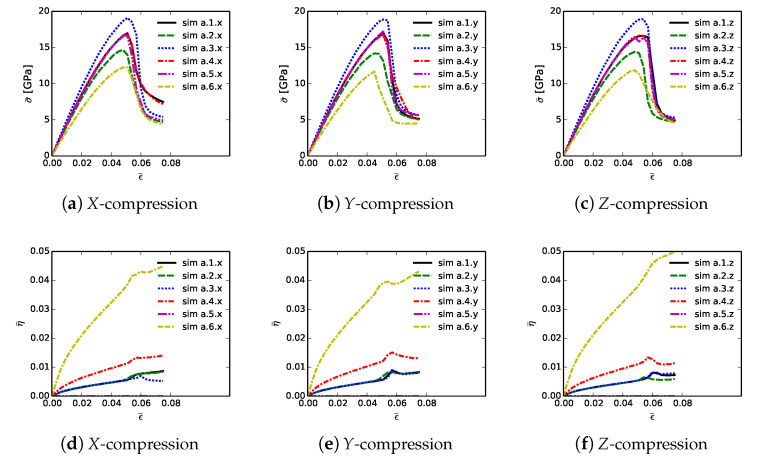
Effective average (Mises) stress $\bar{\sigma }$ and average twin density $\bar{\eta }$ among phase field simulations of Table 2: (**a**) stress, simulations a.1.x through a.6.x, (**b**) stress, simulations a.1.y through a.6.y, (**c**) stress, simulations a.1.z through a.6.z, (**d**) twinning, simulations a.1.x through a.6.x, (**e**) twinning, simulations a.1.y through a.6.y, (**f**) twinning, simulations a.1.z through a.6.z.

Shown in Figure 6 are volume-averaged quantities calculated according to (32) and (33), for all 18 simulations listed in Table 2. Results are plotted versus axial compressive strain $\bar{\epsilon }$ of (31). Simulations are terminated at maximum applied strains of $\bar{\epsilon }=0.075$ due to restrictions on computer wall-clock time and slower numerical convergence in the strain softening regime. However, extending simulations to larger strains would provide no insight into peak strength and ductility, since the polycrystalline aggregates have already surpassed peak loading and undergone load release commensurate with massive fracture. Average stresses are reported in parts (a), (b), and (c), for respective loading directions *X*, *Y*, and *Z*. For uniaxial stress compression loading, effective average stress $\bar{\sigma }$ and the magnitude of average axial stress $\bar{P}$ of (34) are essentially identical. Stress and stiffness are largest for simulations a.3.γ; these contain more diamond than the others at 81.6% by volume. Correspondingly, stress and stiffness are lowest for simulations a.6.γ, which contain less diamond than the others at 39.4%. The next-lowest strength is demonstrated by simulations a.2.γ, which have graphitic layers as the GB phase surrounding large diamond crystals. Differences in average stress–strain behavior are small among the remaining three microstructures, which consider different proportions of SiC single crystals versus SiC matrix material. Furthermore, effects of loading direction and lattice orientation are generally small, since trends and magnitudes do not vary appreciably among parts (a), (b), and (c) of Figure 6.

Shown in Figure 6, parts (d), (e), and (f), is evolution of average twinning order parameter $\bar{\eta }$, which correlates physically with the average volume fraction of twinned material in the microstructure. Recall from Section 3 that diamond does not twin, but single crystals of β-SiC resolved in the simulated microstructure are enabled with twinning capability. Since cases a.4.γ contain no resolved anisotropic SiC single crystals (Table 2), $\bar{\eta }=0$ for these cases. Simulations a.6.γ, followed by a.4.γ, contain more SiC grains by volume than the other cases, so logically these demonstrate the most twinning activity. In cases a.6.γ, the total volume fraction of SiC crystals is 49%, and the twinned volume fraction approaches 5% at $\bar{\epsilon }\approx 0.07$. Thus, around 10% of the SiC granular volume has twinned in these cases.

Table 3 lists mean simulation data at the point of maximum average compressive stress: peak stress ${\bar{P}}_{C}$, axial compressive strain or ductility at this stress level ${\bar{\epsilon }}_{C}$, and cumulative stress work ${\bar{w}}_{C}$ of (34) at applied strain $\bar{\epsilon }={\bar{\epsilon }}_{C}$. Data on each row of this table correspond to the arithmetic mean of values calculated for each of the three loading directions and lattice orientation distributions for each of the six microstructures. Furthermore, reported is the variation $\Delta {\bar{P}}_{C}$, computed as the magnitude of the difference between min and max values among each set of three simulations x,y,z corresponding to a given row in the table.

Trends in ${\bar{P}}_{C}$ are identical to those discussed already for effective stress $\bar{\sigma }$ in the context of Figure 6. Baseline peak unconfined strengths are 16.91 GPa for simulations a.1.γ, at applied strain of 5.2%, and with cumulative energy density of 0.517 GJ/m${}^{3}$. Graphite in cases a.2.γ causes a reduction in ${\bar{P}}_{C}$ by 2.5 GPa, a decrease in average ductility by 0.5%, and a decrease in ${\bar{w}}_{C}$ by over 0.1 GJ/m${}^{3}$. Conversely, the added diamond in cases a.3.γ gives an increase in peak strength by around 2 GPa, with the same ductility, and an increase in ${\bar{w}}_{C}$ of 0.06 GJ/m${}^{3}$. Peak load ${\bar{P}}_{C}$, strain ${\bar{\epsilon }}_{C}$, and energy ${\bar{w}}_{C}$ decrease only slightly from the baseline microstructure while proceeding to cases a.4.γ and then a.5.γ. Variability in peak load due to different microstructure orientations is modest, typically on the order of 0.5 GPa, or around 3% of ${\bar{P}}_{C}$. Simulations with diamond material at GB layers have lowest variability, while those with high SiC matrix content have greatest variability. The weakest microstructures, in terms of ${\bar{P}}_{C}$ and energy absorption capability ${\bar{w}}_{C}$, are those of cases a.6.γ, which have much less diamond and more SiC. However, the most brittle in terms of critical strain ${\bar{\epsilon }}_{C}$ are those with graphite, even though they contain more diamond than cases a.6.γ. Thus, the present results show that replacing diamond with more SiC grains compromises peak strength but not ductility. Replacing SiC GB layers with graphite substantially reduces both peak strength and ductility. Replacing SiC GB layers with isotropic nanocrystalline diamond matrix increases strength but not ductility.

## 5. Molecular Dynamics Simulations

In order to account for the heterogeneity of fracture properties within the composite structure, molecular dynamics (MD) is utilized to calculate fracture energy distributions for both the nanocrystalline-SiC and diamond-SiC boundary elements within the simulations. Utilizing the same EBSD scans from which the microstructure was constructed (Figure 1), the relative orientations of both SiC-SiC and diamond-SiC interfaces were determined. For SiC-SiC interfaces, bicrystals were constructed for the 50 most common orientations, by grain boundary segment length, within the scans. The minimum energy structure of these orientations was then used in constructing the fracture energy distribution. For the diamond-SiC interfaces, a single orientation was found to be most dominant, the ${\left\{111\right\}}_{dia}\left|\right|{\left\{111\right\}}_{SiC}$/${\langle 110\rangle }_{dia}\left|\right|{\langle 112\rangle }_{SiC}$, which is consistent with previous observations [14]. Thus, for these elements, the fracture energies of various metastable interface structures [76] within this orientation were used to create the sampled distribution.

### 5.1. Atomistic Methods

#### 5.1.1. Interface Construction

The orientation information provided by EBSD allows for a definition of the five macroscopic degrees of freedom of the interfaces which describe the relative orientations of the crystals. However, it is still necessary to optimize the atomic configuration so that the structures considered, and the associated fracture energies, correspond to those of the minimum or near-minimum energy states of the interface. To this end, a Monte Carlo approach to interface optimization [77] was employed.

Within this algorithm, and for a given atomic configuration at an interface, three possible operations are considered for each trial move: the insertion of an atom, the removal of an atom, and the switching of the chemical species of the atom between Si and C. Each type of operation occurs with a $\frac{1}{3}$ probability. Potential insertion locations are defined as the vertices of the Voronoi cells formed by the atoms at the interface, with the probability of any specific site being chosen as a function of the site’s distance to the nearest atom, $r_{s}$. For both the removal and switching of chemical species operations, all atoms with non-bulk crystal per-atom energy or local atomic structure, as defined by the common neighbor parameter (CNP), are considered possible sites. The probability of a specific site being operated upon is a function of the difference between its energy and CNP and those of found further from the interface within the bulk crystal. A deeper discussion of the probability functionals, and the Monte Carlo algorithm in general, is available [78].

Once the specific operation and site are determined, energy of the new interfacial atomic structure is rapidly minimized in a three-part process. First, it is quenched from 0.9T${}_{m}$ to 5 K, followed by a thermal equilibration at 5 K, and finally a 0 K conjugate gradient minimization is performed. The energy of the interface is then calculated, and if the operation results in a lower energy structure, the atomic configuration is accepted as the new interfacial structure. If the energy increases, a Metropolis-like criterion accepts or rejects the move based on the resultant change in interfacial energy. This process is repeated 10,000 times for each considered orientation to determined the minimum energy structure, with the added benefit of sampling numerous metastable states in the process.

In order to account for non-stoichiometric interfaces when calculating energetics, the grand canonical potential is utilized [79], with the interfacial energy defined as

(35) $$\begin{array}{c} \gamma_{GB}=\frac{E_{tot}-NE_{SiC}}{A_{GB}}-\frac{\Delta N\Delta \mu }{2A_{GB}}, \end{array}$$

where $E_{tot}$ is the total energy of the system, $E_{SiC}$ the per-atom energy of SiC, $A_{GB}$ the area of the grain boundary, $N=N_{Si}+N_{C}$, and $\Delta N=N_{Si}-N_{C}$, with *N* representing the number of the associated atom type. Quantity Δμ is $\mu_{Si}-\mu_{C}$, where is the chemical potential. However the values of $\mu_{Si}$ and $\mu_{C}$ vary with the chemical environment of the interface. For the SiC-SiC interfaces the chemical environment is assumed to be stoichiometric, and so the midpoint of the possible range of values is used, −2.74 eV. For the diamond-SiC interfaces the chemical environment is carbon-rich, resulting in Δμ=−2.06 eV.

#### 5.1.2. Fracture Energy Calculation

From the constructed interfaces, the fracture energy of each is determined through the strain energy release of the interface at decohesion [80]. Each interface is uniaxially deformed in the tensile direction until failure at a rate of 10${}^{10}$ s${}^{-1}$ and a temperature of 5 K using a modified Nosé-Hoover thermostat. Strain rate studies considering rates of 10${}^{8}$ s${}^{-1}$ and 10${}^{9}$ s${}^{-1}$ were also performed, and results varied by less than 5% from those of 10${}^{10}$ s${}^{-1}$. The highest strain rate was used for computational efficiency. The stress state of the system was determined using the virial theorem [81]. From the resultant stress–strain data and the volume to interface area ratio (*h*), the fracture energy per unit interfacial area was then determined:

(36) $$\Upsilon =\frac{h}{2}\int_{0}^{\epsilon_{f}}\sigma \;d\epsilon .$$

This approach allows all potential fracture paths within the interface to be considered simultaneously, at low computational cost. This makes it feasible to consider a larger number of structures, thus allowing for more robust fracture energy distributions. All MD simulations were performed using the LAMMPS molecular dynamics code [82], using a Si-C interatomic potential developed by Pastewka et al. [83]. This potential is a variant of the Erhart-Albe Tersoff potential [57] with an added screening function [84]. Validation of the potential versus experimental and DFT data for cohesive energies, lattice parameters (i.e., crystal structure), elastic constants, and surface energies of diamond, silicon, graphite, and 3C SiC is described in [83].

The Pastewka potential [83] was chosen due to its ability to successfully model brittle transgranular fracture in both silicon carbide and diamond, with calculated strengths and fracture energies which agree sufficiently with those predicted by DFT in both materials. Results from this MD potential for cohesive stress versus separation distance of (100), (110), and (111) planes match DFT data, where response curves are compared in Figure 4 of [83] for C, Si, and SiC. Theoretical maximum tensile strength of 42 GPa for Σ9[110](122) symmetric tilt grain boundaries in SiC from DFT [85,86] is predicted to within 10% by the Pastewka potential [87].

### 5.2. Results: Sic-Sic Grain Boundaries

**Figure 7:**
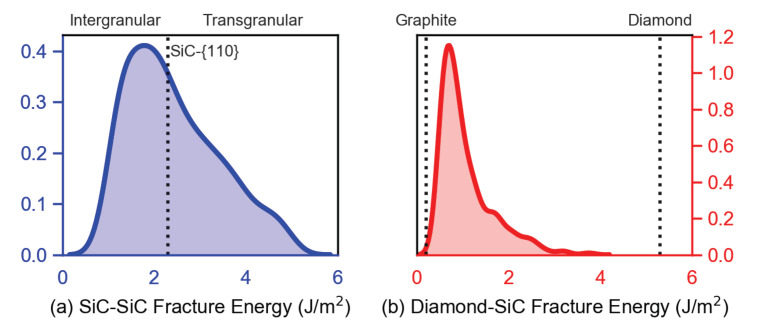
Sampled fracture energy distributions for (**a**) SiC-SiC grain boundaries and (**b**) diamond-SiC interfaces.

Figure 7a shows the resultant distribution of fracture energies from the 50 grain boundaries orientations considered for this work. This distribution is in excellent agreement with both the experimentally determined range of fracture energies for polycrystalline SiC [88] as well as past computational work on SiC grain boundaries [87,89]. Roughly half of the values within the distribution are greater than that of the {110} plane within SiC (2.3 J/m${}^{2}$), which is the preferred cleavage plane and fracture energy of the micro-crystalline SiC (Section 3.1.2). The mean value of Υ for SiC-SiC GBs among structures considered is 2.33 J/m${}^{2}$, and the median value of Υ for SiC-SiC GBs is 2.06 J/m${}^{2}$. Thus, depending on the sampled value of each element, the nanocrystalline SiC matrix can either be stronger or weaker than the SiC micro-crystals within the composite structure. This allows for both transgranular and intergranular crack growth to occur within the phase field simulations, as elements with higher fracture energies may cause the crack propagation through the SiC micro-crystals to become more energetically favorable. As a result, simulations are better able reproduce experimental observations, as both types of fracture behavior (i.e., inter- and transgranular) can occur within SiC [90]. Among the considered GBs, fracture energy was found most correlated with alignment of the interfacial plane to low index planes within the adjacent SiC crystals, particularly {110}, as well as the resolved stress on the 〈110〉{110} direction-plane combination. This is consistent with past work [91], where these were found to be the preferred cleavage plane and the preferred crack propagation direction, respectively, within SiC.

### 5.3. Results: Diamond-Sic Grain Boundaries

**Figure 8:**
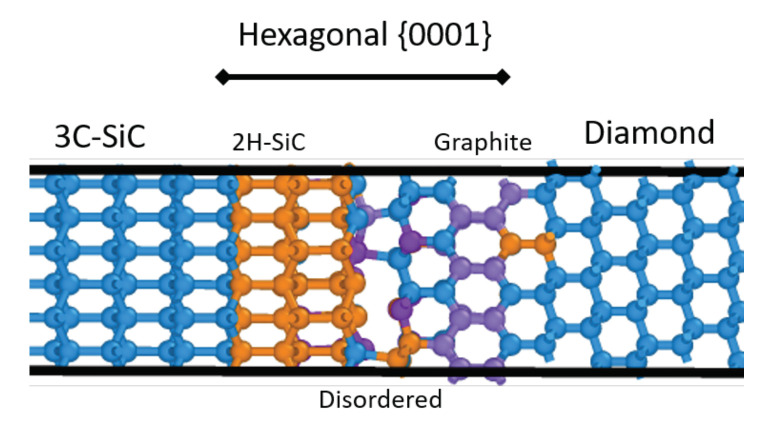
Minimum energy diamond-SiC interface structure as determined by the utilized Monte Carlo algorithm. The atomic configuration is consistent with high resolution transmission electron microscopy [14].

For the diamond-SiC boundary phase elements, approximately 500 metastable states of the ${\left\{111\right\}}_{dia}\left|\right|{\left\{111\right\}}_{SiC}$ /${\langle 110\rangle }_{dia}\left|\right|{\langle 112\rangle }_{SiC}$ orientation were considered. The resultant structures (Figure 8) were found to be consistent with those proposed by Matthey et al. [14], with a {0001} oriented region separating the SiC and diamond phases. Within the MD simulations, failure was found to almost exclusively occur within the SiC region adjacent to the diamond. This is likely a result of the local stress field produced by the non-coherent interface between the two phases [92], in conjunction with the tensile residual stress state of the SiC within these composites [93].

The calculated distribution of fracture energies Figure 7b is lower than that of the SiC grain boundaries, and it is bounded by the fracture energy Υ of pure graphite (0.2 J/m${}^{2}$) and of pure diamond (5.3 J/m${}^{2}$). The mean value of Υ for diamond-SiC boundaries among structures considered is 0.84 J/m${}^{2}$, and the median value of Υ for SiC-SiC GBs is 0.66 J/m${}^{2}$. This suggests that diamond-SiC GBs, even without graphite or voids, are weak links in the composite. Recent micropillar compression experiments on diamond-SiC composites [unpublished results, the authors (J. Ligda et al.)] measured compressive failure strengths of 8 GPa, which are lower than values of 10 GPa measured for 3C-SiC micropillars [71], further supporting (but not proving, since strengths could be influenced by other factors) this hypothesis, since 3C-SiC crystals have much lower fracture energy (cleavage and SiC-SiC GBs) than isolated diamond crystals.

## 6. Phase Field Simulations: Grain Boundary Energy Distribution Effects

Phase field simulations with results in Section 4 assumed fractures were primarily transgranular, whereby fracture surface energy Υ was assigned uniformly among grains or regions belonging to a particular phase in the microstructure: diamond crystals (anisotropic fracture), SiC crystals (anisotropic), SiC matrix (isotropic), diamond matrix (isotropic), or graphite layers (isotropic). In these prior results, Υ was chosen appropriately based on the fracture energy of lowest-energy planes for each crystal type. In contrast, in the present simulations, fractures in material elements of matrix regions are assumed to be intergranular, i.e., along SiC-SiC grain boundaries [19,69] or diamond-SiC phase boundaries [13]. Fracture energy Υ is assigned to the appropriate material phase based on distributions of GB energies calculated from MD in Section 5. Those SiC matrix regions not bordering diamond crystals are nominally assumed to fracture by separation along SiC-SiC grain boundaries [19,69]. Matrix regions of SiC contiguous to diamond are assumed to fracture by separation along diamond-SiC phase boundaries [13]. Fractions of SiC-SiC versus diamond-SiC boundaries vary among microstructures addressed in simulations.

Fracture along diamond-diamond GBs is not quantified in the phase field representation for two reasons. Firstly, in the present diamond-SiC material system, diamond-diamond GBs are very rare, since the microstructure consists of single crystals of diamond usually encased in a SiC matrix, diamond grains bordering larger SiC crystals, or diamond grains coated or in contact with graphitic layers or inclusions. Secondly, experiments [22,94,95] and atomic simulations [96] indicate that polycrystalline diamond tends to fail by transgranular fracture, most often on {111} planes, rather than by intergranular fracture along diamond-diamond GBs.

### 6.1. Simulation Protocols

Eighteen simulations are conducted, six loaded in each of the global *X*, *Y*, and *Z* directions under uniaxial compressive stress conditions (31). Details are listed in Table 4. All simulated microstructures contain the identical assortment of diamond, comprising 70% of the total volume of the composite. The GB layer phase encasing the larger diamond crystals (11.6% by volume) is partitioned into 170 regions, with each discrete region encasing a single diamond crystal. In all 18 of these simulations, a different value of Υ is prescribed to each SiC matrix region, leading to a spatially heterogeneous distribution of fracture strength. Distributed values are assigned consistently with MD results of Section 5.2 that consider a realistic variety of SiC-SiC GB types. Different realizations are enacted for each of the 18 simulations, i.e., a different spatial distribution of GB energy is invoked in each simulation, though all statistically correlate with probability distribution functions constructed from MD solution data of Section 5.2. Simulations require 177 or 355 distinct values of Υ for SiC-SiC GB fracture depending on the number of simulated SiC matrix regions. Simulations b.1.γ, b.2.γ, b.5.γ, and b.6.γ are assigned discrete values of Υ to each of the 170 GB regions in an analogous way, but with diamond-SiC GB energies from Section 5.3 used rather than SiC-SiC GB energies. Simulations b.3.γ and b.4.γ contain graphite rather than SiC in GB layers surrounding diamond crystals. Here, fracture energy Υ for the 170 layered regions is assigned as that of basal planes of graphite, since these are presumed much weaker than any other grain or phase boundary (Table 1). Simulations b.2.γ, b.4.γ, and b.6.γ assume failure in SiC matrix regions is dominated by phase boundaries between these regions and diamond grains in close proximity.

Simulations b.5.γ and b.6.γ have more regions of nanocrystalline SiC matrix (355 versus 177) in which failure may occur by GB decohesion, so effects of MD energy distributions are expected to be greater for these simulations relative to cases with β≤4. Cases b.$\beta_{1}$.$\gamma_{1}$ have the same lattice orientations (S,M,N; Roman numerals I–IX in Table 4) for bulk grains as cases b.$\beta_{2}$.$\gamma_{2}$ when $\gamma_{1}=\gamma_{2}$ and $\beta_{1}=1,2$ and $\beta_{2}=1,2$, or $\beta_{1}=3,4$ and $\beta_{2}=3,4$, or $\beta_{1}=5,6$ and $\beta_{2}=5,6$. For these matching paired cases, microstructure parameters differ only with regard to discrete instantiations of fracture energies (numbered 1–18) obtained from MD simulations. Such prescriptions enable effects of GB fracture energies to be distinguished from effects of orientation distributions of cleavage and twinning planes.

### 6.2. Results

Simulations listed in Table 4 consider different GB fracture property distributions among numerous regions of matrix material and layers encasing larger grains. Effects on model predictions are discussed next. Displacement boundary conditions are the same as those of Section 4: normal strain $\bar{\epsilon }\geq 0$, defined in (31), compresses the aggregate in one of global *X*, *Y*, or *Z* directions.

**Figure 9:**
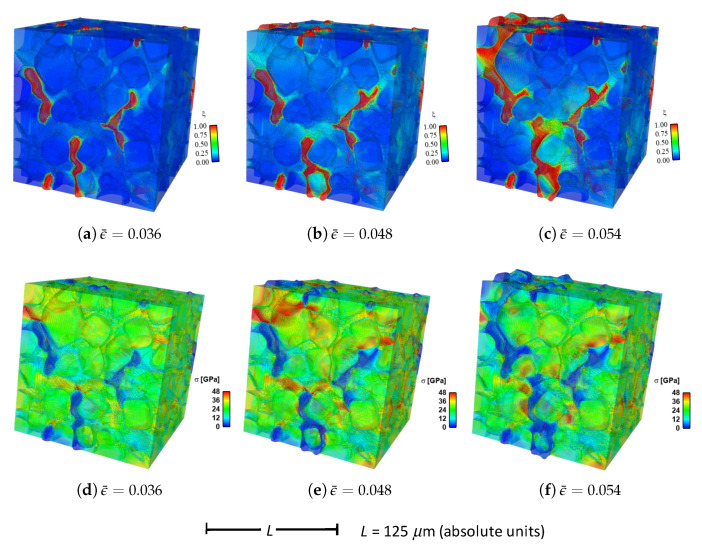
Stress and fracture order parameter fields for simulation b.6.z (70% diamond, mixed SiC grains and matrix, SiC GB layers, MD distributions for boundary fracture energies): (**a**) fracture order parameter ξ, $\bar{\epsilon }=0.036$, (**b**) fracture order parameter ξ, $\bar{\epsilon }=0.048$, (**c**) fracture order parameter ξ, $\bar{\epsilon }=0.054$, (**d**) effective stress σ, $\bar{\epsilon }=0.036$, (**e**) effective stress σ, $\bar{\epsilon }=0.048$, (**f**) effective stress σ, $\bar{\epsilon }=0.054$.

Given in Figure 9 are contours of effective stress and order parameter fields for simulation b.6.z, compressed along the *Z*-direction of Figure 3. This microstructure contains 70% total fraction of diamond, 30% β-SiC. The SiC is divided into isotropic matrix (355 regions, 18.4%) and GB layers (170 regions, 11.6% by volume) encasing the large diamond crystals. Differences between the current case and that shown in Figure 4 include the current use of distributions of fracture energies for distributions of energies for diamond-SiC interfaces (from MD results of Section 5.3). In the former case of Figure 4, uniform fracture energies for SiC cleavage on {110} planes were assigned instead. Fractures most commonly commence in the SiC matrix phase intermixed with smaller diamond crystals, in particular regions where assigned interfacial strengths are low. Dominant cracks, and cleavage fractures in diamond, are more scarce than those reported for simulations in Section 4. Stresses appear most often largest in the stiffer diamond phase, and are highly relaxed where fractures occur.

**Figure 10:**
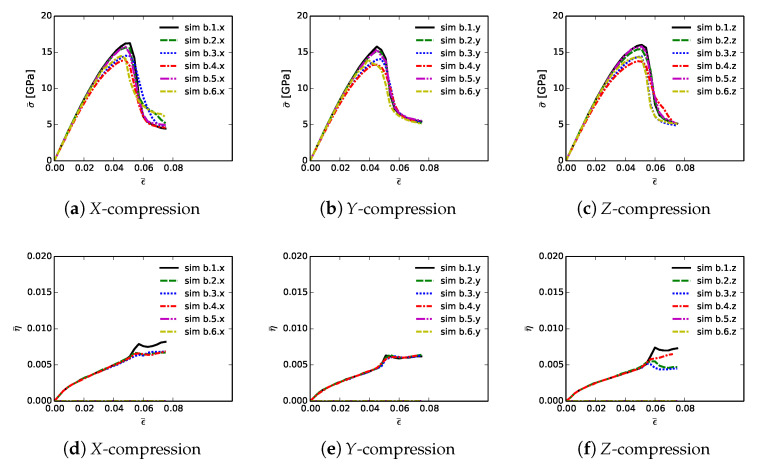
Effective average (Mises) stress $\bar{\sigma }$ and average twin density $\bar{\eta }$ among phase field simulations of Table 4: (**a**) stress, simulations b.1.x through b.6.x, (**b**) stress, simulations b.1.y through b.6.y, (**c**) stress, simulations b.1.z through b.6.z, (**d**) twinning, simulations b.1.x through b.6.x, (**e**) twinning, simulations b.1.y through b.6.y, (**f**) twinning, simulations b.1.z through b.6.z.

Volume averages of solution variables are shown in Figure 10. These are computed according to (32) and (33), for all 18 simulations listed in Table 4, and are plotted versus axial compressive strain $\bar{\epsilon }$ of (31). Average effective stresses are given in parts (a), (b), and (c), for respective loading directions *X*, *Y*, and *Z*. Average stress are usually largest for simulations b.1.γ up until peak loading. These contain more discrete SiC crystals than b.5.γ and b.6.γ, so are less prone to fail by GB decohesion at grain/phase boundaries. Lowest strength and tangent stiffness are demonstrated by cases b.4.γ, which contain graphite as the GB layer material and a high fraction of diamond-SiC boundaries controlling matrix failure. Fracture energy of graphite used in simulations (weak basal planes) is notably lower than the average fracture energy of SiC-SiC and diamond-SiC boundaries. Additionally, diamond-SiC boundaries are weaker than SiC-SiC boundaries (Section 5), explaining lower compressive strengths of cases b.$\beta_{2}$.γ relative to b.$\beta_{1}$.γ, where $\beta_{2}=2,4,6$ and $\beta_{1}=1,3,5$. Differences in average stress–strain behavior are very small among cases with the same lattice orientation distributions and area fractions of SiC-SiC versus SiC-diamond boundaries, but with different grain and phase boundary energy distributions. Effects of loading direction and lattice orientation are detectable, but still small, upon comparison of like simulations b.β.γ in parts (a), (b), and (c) of Figure 10.

Average twinning parameter $\bar{\eta }$ is shown in Figure 10, parts (d), (e), and (f). This is physically related to the average volume fraction of twinned material in the composite, noting from Section 3 that diamond does not twin, but single crystals of β-SiC may twin in simulations depending on orientation and stress state. Results shown here are similar to those for cases a.1.γ and a.2.γ in Figure 6. Magnitudes are sensitive to initial lattice orientation, and thus loading direction, with cases b.β.y in Figure 10e exhibiting generally lower average $\bar{\eta }$ than the other directions in Figure 10d,f. Average twinned fraction does not vary with GB fracture energy distribution at strains up to peak load, $\bar{\epsilon }≲0.05$. Subsequently at larger strains, heterogeneous deformation and stress fields induced by fractures lead to different twinning behaviors among different cases in each of parts (d), (e), and (f) of Figure 10.

Table 5 contains mean simulation data at the point of maximum average compressive stress: peak stress ${\bar{P}}_{C}$, axial compressive strain, i.e., ductility, at this stress level denoted ${\bar{\epsilon }}_{C}$, and cumulative stress work ${\bar{w}}_{C}$ defined generally in (34) at applied strain $\bar{\epsilon }={\bar{\epsilon }}_{C}$. Data on each row correspond to the arithmetic mean of values for each of the three loading directions and orientation distributions for each of the six microstructures. Variations of peak normal stress among each of the three directions are also listed in each row.

Trends in ${\bar{P}}_{C}$ match those of effective stress $\bar{\sigma }$ reported in the context of Figure 10. Cases b.1.γ differ slightly due only to different GB fracture energy distributions, with average and variations of values of peak strength of 16.06 and 0.50 GPa, respectively. These are compared to baseline peak unconfined strength 16.91 GPa, with variation 0.39 GPa, for simulations a.1.γ, which assumed uniform toughness of matrix phases corresponding to transgranular fractures. Use of distributed energies for intergranular fractures therefore leads to a decrease in peak average strength and an increase in variability $\Delta {\bar{P}}_{C}$. Ductility ${\bar{\epsilon }}_{C}$ and cumulative work ${\bar{w}}_{C}$ are also reduced: 0.052 (a.1.γ) versus 0.049 (b.1.γ) and 0.517 GJ/m${}^{3}$ (a.1.γ) versus 0.464 GJ/m${}^{3}$ (b.1.γ). Graphite in simulations b.3.γ and b.4.γ reduces ${\bar{P}}_{C}$ by around 1.7 GPa and decreases ${\bar{w}}_{C}$ by around 0.05 GJ/m${}^{3}$. These reductions, though less severe, are consistent with those observed previously in Table 3. Unconfined compressive strength ${\bar{P}}_{C}$, strain ${\bar{\epsilon }}_{C}$, and energy ${\bar{w}}_{C}$ are slightly reduced in cases b.5.γ and b.6.γ. Recall that these cases contain more SiC matrix regions, and hence more material susceptible to intergranular fracture than baseline simulations b.1.γ and b.2.γ. The current results again show that replacing SiC GB layers with graphite substantially reduces both peak strength and ductility, consistent with some experiments [16,17] that indicate deleterious effects of graphite. Furthermore, when matrix failure is affected by non-uniform distributions of GB fracture strengths, overall integrity of the composite is reduced relative to simulations in which local GB fracture energies are uniformly assigned.

## 7. Phase Field Theory: Defective Matrix and Gb Regions

The model of Section 2, Section 3 and Section 5 is extended to account for processing defects, namely graphitic inclusions and pores. Graphite is assumed to present itself in the form of thin layers or lamellae between fine matrix grains, especially in the vicinity of SiC-diamond and SiC-SiC interfaces [14,15,16]. Pores also tend to arise in the vicinity of grain and phase boundaries, rather than within individual crystals. Physical dimensions of these entities are typically on the order of 10 nm, though sizes may vary between several and several hundred nm. These defects are around two orders of magnitude smaller than the bulk diamond grain size $l_{G}$ [12,13,14,15]. Residual silicon (Si) can also appear in small inclusions depending on processing, but is omitted in the present study.

In the current context, resolution of discrete defects (graphitic nano-layers, inclusions, and voids) is intractable using phase field theory when the entire domain contains numerous larger single crystals. Exceedingly refined FE meshes with an immense number of elements would be required. Instead, properties are homogenized for representative isotropic materials that address defect content in an average sense within thicker resolved layers surrounding bulk grains as well as the nanocrystalline matrix. These representative materials, in fully dense form, are assigned effective elastic constants based on properties and volume fractions of their solid constituents tabulated in Section 3. Effective surface energies for fracture at SiC-SiC or SiC-diamond interfaces within representative mixtures are obtained from Section 5. Finally, homogenized properties for elasticity and fracture strength are degraded according to the initial void volume fraction, if nonzero. Specifically, the effective isotropic bulk and shear moduli are reduced with porosity using the self-consistent theory of [97], which is further discussed in [98,99]. The fracture surface energy is reduced proportionally to the effective elastic modulus, following the empirical model and observations from [100,101,102].

This homogenized material (crystalline SiC matrix + fine-scale GB interfaces + graphite + pores) is herein referred to simply as the “effective material”. The present approach parallels that introduced previously for defective GB regions in [23]. A comprehensive account of the model features outlined in Section 7.1, Section 7.2 and Section 7.3 can be found in that reference [23], including a physical description of microstructure features and length scales, derivations of averaging methods, and justifications and limitations of the model. Notably, this homogenization scheme for effective properties produces effective moduli and fracture energy that depend explicitly only on the known properties and volume fractions of the constituents, without recourse to ad hoc parameters.

Different microstructures of diamond-SiC composites can be achieved depending on choices and proportions of starting materials and processing routes [10,11,12,13,14,15,16,103]. Theoretically dense materials can be produced via infiltration of inter-crystalline regions by the matrix phase [11,16]. In materials with finite porosity [13,103], voids are usually found at local grain and phase boundaries in the matrix, rather than within bulk single crystals. Graphite may present itself in layered or agglomerated forms [14,16,69,104]. Ranges of porosity and graphite fractions investigated computationally (i.e., up to 8% of total volume) are motivated by percentages reported elsewhere for various diamond-SiC composites [13,14,16,103]. Although higher porosities may arise in other ceramics [102], the maximum porosity realistically modeled here is capped by limits of elastic homogenization in Section 7.2, e.g., with theoretical error on degraded shear modulus on the order of the square of porosity [97].

### 7.1. SIC-Graphite Solid Mixture

The local volume fraction of solid material in the effective material occupied by graphite is denoted by $\upsilon_{g}$, such that $\upsilon_{s}=1-\upsilon_{g}$ is the solid fraction of cubic nanocrystalline SiC. Denote by ($k_{s},\;k_{g}$) the isotropic bulk moduli $k_{0}$ of (SiC, graphite) phases. Denote by ($\mu_{s},\;\mu_{g}$) the isotropic shear moduli $\mu_{0}$ of (SiC, graphite). Effective isotropic elastic constants for this mixture are the arithmetic average of upper and lower Hashin-Shtrikman (H-S) type bounds for two-phase composites [105]:

(37) $$\begin{array}{cc} {\hat{k}}_{0} & =\frac{1}{2}\left[k_{g}+\frac{\upsilon_{s}}{1/\left(k_{s}-k_{g}\right)+3\upsilon_{g}/\left(3k_{g}+4\mu_{g}\right)}+k_{s}+\frac{\upsilon_{g}}{1/\left(k_{g}-k_{s}\right)+3\upsilon_{s}/\left(3k_{s}+4\mu_{s}\right)}\right];\\ {\hat{\mu }}_{0} & =\frac{1}{2}\left[\frac{1}{2R_{u}}-\frac{9}{4}k_{s}+\frac{1}{2R_{l}}-k_{g}\right];\\ R_{u} & =\frac{\upsilon_{s}}{2\mu_{s}+\left(9/2\right)k_{s}}+\frac{\upsilon_{g}}{2\mu_{g}+\left(9/2\right)k_{s}},\;R_{l}=\frac{\upsilon_{s}}{2\mu_{s}+2k_{g}}+\frac{\upsilon_{g}}{2\mu_{g}+2k_{g}}. \end{array}$$

Numerical values of $\mu_{0}$ and $k_{0}=\lambda_{0}+\frac{2}{3}\mu_{0}$ from Table 1 are inserted into (37) to yield ${\hat{k}}_{0},{\hat{\mu }}_{0}$ for the solid mixture that depend uniquely on $\upsilon_{g}=1-\upsilon_{s}$. Elastic constants are assumed unaffected by grain boundaries. As shown in [23], the average of the H-S bounds for this material system is nearly identical to the Hill average, so the latter (whose derivation does not imply isotropy of each individual phase) could be used instead without consequence.

Upper and lower bounds for $\hat{\Upsilon }$, the effective fracture surface energy for the solid mixture, are estimated as respective Voigt and Reuss averages:

(38) $${\hat{\Upsilon }}_{u}=\upsilon_{s}\Upsilon_{s}+\upsilon_{g}\Upsilon_{g},\;{\hat{\Upsilon }}_{l}={\left[\upsilon_{s}/\Upsilon_{s}+\upsilon_{g}/\Upsilon_{g}\right]}^{-1}.$$

Subscripts ${\left(\cdot \right)}_{s}$ and ${\left(\cdot \right)}_{g}$ correspond to properties of the SiC phase and graphite, respectively. Fracture surface energy of SiC can be calculated in one of three ways, depending on the location and composition of the material element in question. The first way, as used in [23], invokes Υ from Table 1, where weak links are assumed to be {110} cleavage planes. The second way invokes either the mean or distribution of Υ as calculated in Section 5.2, assuming weak links are SiC-SiC grain boundaries. Since the mean GB energy is coincidentally equal to the {110} cleavage energy for 3C-SiC, both assumptions provide the same result when uniform properties are assigned. On the other hand, when distributions of SiC-SiC GB fracture energies are imposed as in Section 6, the two assumptions yield different local properties for each element of effective material. The third way assumes SiC-diamond boundaries are weak links in the matrix phase in the absence of graphite, whereby the mean or distribution of Υ as calculated in Section 5.3 is used, depending on whether uniform or heterogeneous GB strengths are applied. This third method would realistically apply for regions of effective material in close proximity to diamond grains, where behavior of phase boundaries dominates. Such is the case for the present material system, suggested by much weaker diamond-SiC boundaries than SiC-SiC boundaries and SiC cleavage planes according to MD results of Section 5.

Importantly, $\Upsilon_{g}\ll \Upsilon_{s}\Rightarrow {\hat{\Upsilon }}_{l}\ll {\hat{\Upsilon }}_{u}$. The upper bound in (38) was used to represent spherical graphitic inclusions in a recent phase field study [23]. In that work, effects of graphite on predicted failure behavior were relatively small for volume fractions $\upsilon_{g}≲0.04$. In the present work, the lower bound ${\hat{\Upsilon }}_{l}$ is used, as it is thought to be more applicable to address effects of thin layers of graphite that more drastically degrade material integrity. For example, reductions in indentation hardness of 20 to 30 GPa were observed [16] for a diamond-SiC composite containing ≈0.5–5 % graphite in layered form concentrated around diamond crystals.

In this paper, graphite is treated as an elastic material that may fracture on weak (0001) planes. Phase transformations, plasticity, and sliding mechanisms that could lead to toughening under high confinement, pressure-shear conditions [19] (e.g., indentation) are not included since maximum pressures attained under the present uniaxial stress loading conditions are much lower. Prescription of a fracture energy increasing with defect fractions would provide increased integrity of the composite as shown in [23], contradicting many, but not all, experimental results.

### 7.2. Porosity

Voids degrade elastic properties following a self-consistent scheme [97,98,99]. Let $\varphi =1-\rho /\rho_{0}$, within possible range 0≤φ<1, represent the pore volume fraction, with ρ the mass density of the undeformed material with voids and $\rho_{0}$ the mass density of the undeformed solid without voids. Denote by ${\hat{k}}_{0}$, ${\hat{\mu }}_{0}$, and ${\hat{\nu }}_{0}$ the bulk modulus, shear modulus, and Poisson’s ratio of the solid of density $\rho_{0}$, computed according to (37). Let ${\bar{k}}_{0}$, ${\bar{\mu }}_{0}$, and ${\bar{\nu }}_{0}$ denote, respectively, the effective bulk modulus, shear modulus, and Poisson’s ratio of the material with voids, of density ρ. Then, following [97,99],

(39) $${\bar{k}}_{0}={\hat{k}}_{0}\left[1-\frac{3(1-{\hat{\nu }}_{0})\varphi }{\left(1+{\hat{\nu }}_{0}\right)\varphi +2\left(1-2{\hat{\nu }}_{0}\right)}\right],\;{\bar{\mu }}_{0}={\hat{\mu }}_{0}\left[1-\frac{15(1-{\hat{\nu }}_{0})\varphi }{7-5{\hat{\nu }}_{0}}\right].$$

The constraints ${\bar{k}}_{0}\geq \zeta {\hat{k}}_{0}$ and ${\bar{\mu }}_{0}\geq \zeta {\hat{\mu }}_{0}$, where ζ=0.01, maintain a nonzero effective stiffness, e.g., if φ is large.

Based on theoretical derivations and experimental observations on various ceramics and brittle solids [100,101], effective fracture surface energy for the porous mixture, $\bar{\Upsilon }$, is assumed to be affected by pores analogously to the elastic modulus of the porous mixture, ${\bar{E}}_{0}$. With Poisson’s ratio and Young’s modulus ${\hat{\nu }}_{0}=\left(3{\hat{k}}_{0}-2{\hat{\mu }}_{0}\right)/\left(6{\hat{k}}_{0}+2{\hat{\mu }}_{0}\right)$ and ${\hat{E}}_{0}=\left(2+2{\hat{\nu }}_{0}\right){\hat{\mu }}_{0}$, and complementary definitions for ${\bar{\nu }}_{0}$ and ${\bar{E}}_{0}$,

(40) $$\begin{array}{cc} \bar{\Upsilon } & ={\hat{\Upsilon }}_{u}\frac{{\bar{E}}_{0}}{{\hat{E}}_{0}}={\hat{\Upsilon }}_{u}\frac{\left(1+{\bar{\nu }}_{0}\right){\bar{\mu }}_{0}}{\left(1+{\hat{\nu }}_{0}\right){\hat{\mu }}_{0}}\;\left(\mathrm{upper}\;\mathrm{bound}\right);\\ \bar{\Upsilon } & ={\hat{\Upsilon }}_{l}\frac{{\bar{E}}_{0}}{{\hat{E}}_{0}}={\hat{\Upsilon }}_{l}\frac{\left(1+{\bar{\nu }}_{0}\right){\bar{\mu }}_{0}}{\left(1+{\hat{\nu }}_{0}\right){\hat{\mu }}_{0}}\;\left(\mathrm{lower}\;\mathrm{bound}\right). \end{array}$$

The lower bound in the second of (40) is used subsequently herein, consistent with remarks in Section 7.1 As discussed in detail in [23], the reduction in fracture energy due to voids in (40) is based on empirical evidence for other ceramics [100,101,102]. It has not been verified for a SiC-graphite composite with nano-voids pertinent to the present simulations, for which experimental toughness data versus defect volume fractions (porosity and graphite) do not exist. In certain cases, considered briefly in prior work [23], pores have been observed to improve, rather than degrade, toughness [102].

### 7.3. Effective Phase Field Properties

**Figure 11:**
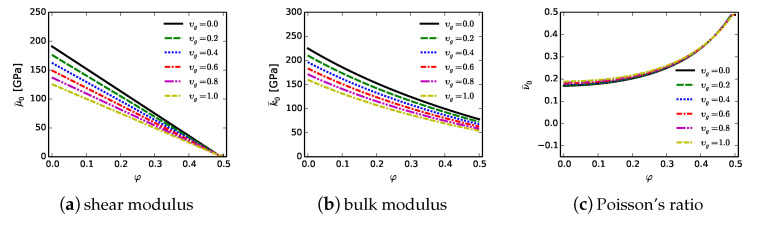
Elastic properties for isotropic material consisting of nanocrystalline SiC, graphite ($\upsilon_{g}$), and/or pores (φ): (**a**) shear modulus ${\bar{\mu }}_{0}$ (**b**) bulk modulus ${\bar{k}}_{0}$ (**c**) Poisson’s ratio ${\bar{\nu }}_{0}$. Parts (**a**,**b**) reproduced from [23].

**Figure 12:**
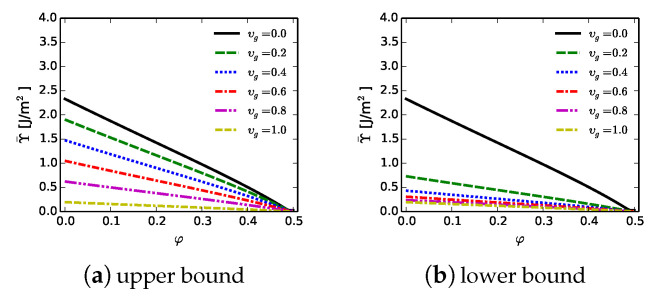
Effective fracture surface energy for GB phase consisting of nanocrystalline SiC, graphite ($\upsilon_{g}$), and/or pores (φ): (**a**) upper bound on $\bar{\Upsilon }$ from (40) (**b**) lower bound on $\bar{\Upsilon }$ from (40). Part (**a**) reproduced from [23].

The phase field framework of Section 2 for bulk crystals, informed by Section 5 for GB fracture energies, is modified slightly to describe effective material regions with sub-scale defects. Elastic (${\bar{\lambda }}_{0},{\bar{\mu }}_{0}$) and fracture ($\bar{\Upsilon }$) properties depend on the initial void volume fraction φ and the solid fraction of graphite $\upsilon_{g}$ according to Section 7.1 and Section 7.2. Ranges of elastic stiffnesses ${\bar{\mu }}_{0}$, ${\bar{k}}_{0}={\bar{\lambda }}_{0}+\frac{2}{3}{\bar{\mu }}_{0}$, and Poisson’s ratio ${\bar{\nu }}_{0}=\left(3{\bar{k}}_{0}-2{\bar{\mu }}_{0}\right)/\left(6{\bar{k}}_{0}+2{\bar{\mu }}_{0}\right)$ from (39) are shown in Figure 11 [23]. Estimated upper and lower bounds on fracture energy $\bar{\Upsilon }$ computed from (40) are shown in Figure 12. Here, Υ=2.33 J/m${}^{2}$ is used for the SiC phase, though in calculations, ranges are possible depending on which of the assumptions for Υ are invoked from Section 7.1. Fracture energy $\bar{\Upsilon }$ decreases with increasing graphite fraction. Decreases are more dramatic for the lower bound approximation in Figure 12b, which is invoked subsequently in calculations of Section 8. The rationale is that failure by separation of basal planes in graphite is assumed to dominate the overall fracture energy of the effective material when the graphite manifests in thin layers that percolate throughout grain and phase interfaces.

Since the effective material is idealized as isotropic, $\hat{\beta }=0$. Values of α, χ, and *l* are identical to their counterparts in Table 1, which do not vary with solid constituent. Twinning, by nature a highly anisotropic process, is omitted following reasons outlined in Section 3 for the model of isotropic matrix phases. Cavitation or bulking associated with opening of newly formed fracture surfaces is omitted here in the effective material (i.e., $x_{\xi }=0$) since existing pores provide free volume to accommodate localized expansion, and since graphitic layers provide lubrication to fractured areas whose asperities might otherwise induce expansion upon relative slippage of free surfaces.

## 8. Phase Field Simulations: Distributed Graphite and Porosity Effects

Considered in Section 8 are simulation outcomes for microstructures containing matrix and GB layer phases consisting of mixtures of nanocrystalline SiC, graphite with solid fraction $\upsilon_{g}$ and/or initial porosity with local volume fraction φ. In all cases, a distribution of fracture energies is first assigned to matrix and GB layer regions from MD simulation results on Υ detailed in Section 5. The same strategy discussed in Section 6.1 is used to assign MD values to phase field realizations. Matrix regions (with or without pores and graphitic inclusions) to have a baseline fracture energy distribution corresponding to diamond-SiC GB interfaces, which are the weakest links in these regions of the composite material in the absence of other defects. These energies are then modified according to equations in Section 7, specifically the lower bound model of (40) and Figure 12b, to account for initial voids and/or graphite. Elastic constants in matrix regions containing voids and/or graphite are likewise modified according (37) and (39), shown in Figure 11. Discrete diamond and SiC single crystals are modeled according to descriptions of Section 3.1.1 and Section 3.1.2. These crystals are fully dense and contain no internal graphite; hence, their fractures are transgranular.

### 8.1. Simulation Protocols

Eighteen simulations are performed to interrogate effects of initial pores and distributed graphite, superposed on GB fracture energy distributions prescribed from MD. These are denoted as simulations c.β.γ in Table 6, where β=1,…,6 and γ = x, y, or z depending on the direction of uniaxial stress compressive loading prescribed by (31). Lattice orientations for cleavage fracture and twinning differ among values of γ at fixed β, but are matching among all six cases sharing the same value of γ. The same protocols are enforced for shared distributions of GB fracture energies. All simulations have identical phase distributions apart from graphite and pore content that vary systematically among values of β. Specifically, the diamond fraction is fixed at 70%, anisotropic SiC grains are fixed at 9.4%, isotropic SiC matrix (+ potential defects) is 9.0%, and the GB layer phase (SiC + potential defects) is 11.6%. Simulations c.1.γ contain no initial defects; these are baseline cases for comparison. Simulations c.β.γ, where β=2,…,6, contain uniform solid fractions of graphite $\upsilon_{g}$ and/or porosity φ in matrix and GB layer regions as indicated in Table 6. Values of $\upsilon_{g}$ = 0, 0.2, or 0.4 are prescribed exactly for these local regions, and similarly values of φ = 0, 0.2, or 0.4 are prescribed exactly depending on the particular value of β∈[2,6]. The total volume fraction of a defect type (pores or graphite) in the entire microstructure reported in Table 6 is then calculated from the local defect concentrations and the volume fractions of each phase containing these local concentrations.

### 8.2. Results

Simulations listed in Table 6 consider different initial concentrations of graphitic inclusions and voids among numerous regions of matrix material and layers encasing larger grains. Effects on model predictions are discussed in what follows. Normal strain $\bar{\epsilon }\geq 0$, defined in (31), is imposed on the aggregate in one of the global *X*, *Y*, or *Z* directions.

**Figure 13:**
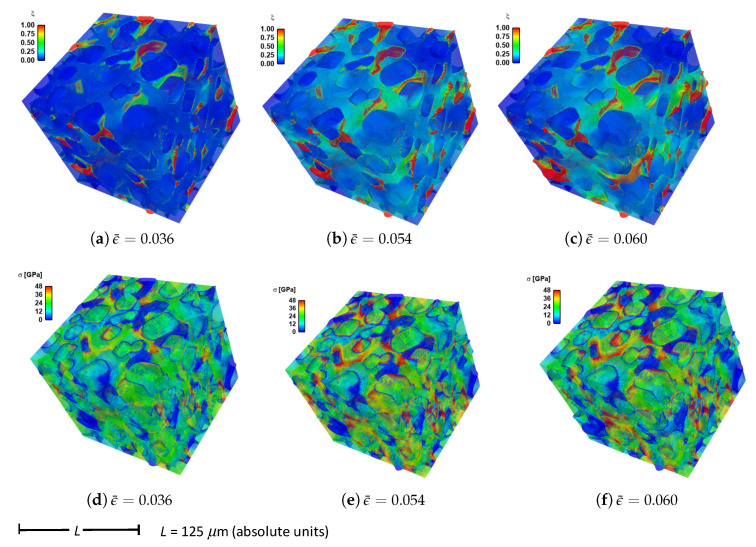
Stress and fracture order parameter fields for simulation c.6.x (70% diamond, mixed SiC grains and matrix, defective SiC GB layers, MD distributions for intergranular fracture energies, $\upsilon_{g}=0.4$, φ=0.4, total graphite fraction 5%, total porosity 8%): (**a**) fracture order parameter ξ, $\bar{\epsilon }=0.036$, (**b**) fracture order parameter ξ, $\bar{\epsilon }=0.054$, (**c**) fracture order parameter ξ, $\bar{\epsilon }=0.060$, (**d**) effective stress σ, $\bar{\epsilon }=0.036$, (**e**) effective stress σ, $\bar{\epsilon }=0.052$, (**f**) effective stress σ, $\bar{\epsilon }=0.060$.

Shown in Figure 13 are von Mises stress σ and order parameters ξ and η for simulation c.6.x, compressed in the global *X*-direction of Figure 3. This microstructure is the most defective of those listed in Table 6. The composite contains 70% diamond by initial volume and 17% β-SiC. Graphite (5% of total volume) and pores (8% of total volume) are distributed homogeneously among the SiC regions of interspersed matrix material and the GB layer material. The SiC is divided into discrete grains (178 crystals) and isotropic matrix with pores and graphite (177 regions). A nanocrystalline SiC matrix with pores and graphite comprises the GB layers (170 regions) encasing the large diamond crystals. Differences between the current case and that shown in Figure 9 are the graphitic inclusions and pores added here that affect elastic and fracture behaviors in regions containing these defects, as modeled according to the theory of Section 7 and [23]. Effective deviatoric stress σ is highly relaxed in defective regions as a result of their much lower shear moduli relative to pure diamond or pure SiC (Figure 11a). Differences between the current case and that of Figure 4 include the aforementioned graphite and pores, as well as the current use of distributions of fracture energies acquired from MD for the matrix and GB layer materials. Fractures initiate in the SiC matrix and GB layer phases in Figure 13, in regions where baseline diamond-SiC interfacial strengths from MD distributions are low and are then further degraded due to graphitic lamellae and voids. Intragranular fractures are scarce. Trends in twin activity (not shown in Figure 13) are similar to prior results in Figure 4 and Figure 5 where only a select few grains twin prominently due to the low overall volume fraction of discrete SiC crystals (9.4%) for which twinning is possible. As was noted in the context of prior results in Section 4 and Section 6, σ is usually largest in the stiffer diamond phase, and smallest where relaxation due to fractures arises.

**Figure 14:**
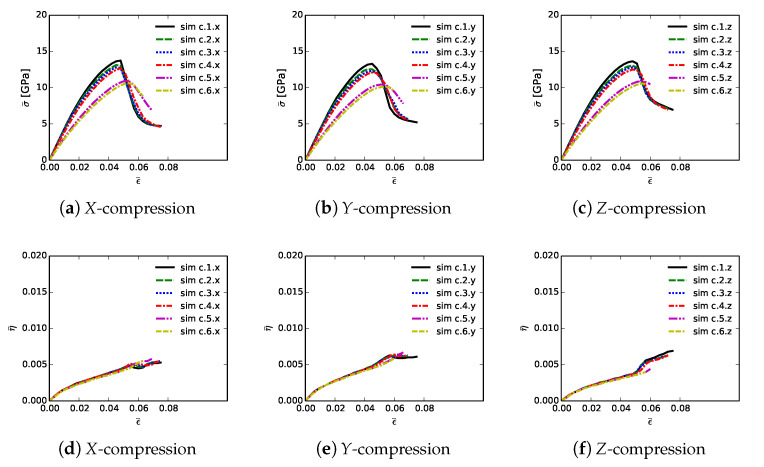
Effective average (Mises) stress $\bar{\sigma }$, average fracture density $\bar{\xi }$, and average twin density $\bar{\eta }$ among phase field simulations of Table 4: (**a**) stress, simulations c.1.x through c.6.x, (**b**) stress, simulations c.1.y through c.6.y, (**c**) stress, simulations c.1.z through c.6.z, (**d**) twinning, simulations c.1.x through c.6.x, (**e**) twinning, simulations c.1.y through c.6.y, (**f**) twinning, simulations c.1.z through c.6.z.

Evolution of average effective stress, fracture order parameter, and twinning order parameter are shown in Figure 14. These are computed via (32) and (33), for all 18 simulations listed in Table 6. Results are plotted versus $\bar{\epsilon }$ of (31).

Average effective stresses are reported in Figure 14, parts (a), (b), and (c), for respective loading directions *X*, *Y*, and *Z*. Average stress are clearly largest for simulations c.1.γ up until peak loading. Recall that cases c.1.γ contain no graphite or pores, so results are similar to cases b.1.γ and b.2.γ in Figure 10, though peak strength is reduced somewhat here due to the present assumption of $x_{\xi }=0$ for GB fractures [23]. The weakest microstructures are those that contain the largest porosity, notably cases c.5.γ (8% porosity) and c.6.γ (8% porosity, 5% graphite). Differences in average stress–strain behavior among different initial lattice orientations and GB energy distributions are detectable, but minor, upon comparison of like simulations c.β.γ in parts (a), (b), and (c) of Figure 14.

Shown in Figure 14 is $\bar{\eta }$, with different loading directions and microstructure orientation distributions shown in parts (d), (e), and (f). Results are similar to those for cases a.1.γ and a.2.γ in Figure 6 and cases b.β.γ (β=1,…,4) in Figure 10. Magnitudes are sensitive to initial lattice orientation that controls Schmid factor for elastic driving force for twinning, and therefore loading direction. At larger strains, differences among individual simulations in parts (d), (e), and (f) of Figure 14 are more pronounced. Slightly less twinning is observed for cases c.β.γ relative to those of Section 4 and Section 6 since lower effective stresses in the presence of pores and graphite lead to lower driving forces.

Table 5 includes mean simulation data at the point of maximum average compressive stress. Data in each row correspond to the arithmetic mean of values for each of the three loading directions, orientation distributions, and GB fracture energy distributions for each of the six microstructures β=1,…,6. Trends in ${\bar{P}}_{C}$ are consistent with those discussed for effective stress $\bar{\sigma }$ already in the context of Figure 14. The baseline case with no initial defects, i.e., null porosity and graphite, is c.1.γ with average strength, ductility, and energy density, respectively, of 13.58 GPa, 0.047, and 0.396 GJ/m${}^{3}$. The reduction in strength due to 4% graphite is 0.64 GPa, and the reduction due to 4% void volume fraction is 1.13 GPa. Increasing graphite fraction from 4% to 8% induces a further reduction in strength by only 0.28 GPa, as evidenced by comparing results for c.2.γ with c.3.γ. Increasing the void fraction from 4% to 8% causes a more drastic strength reduction by another 1.66 GPa, as seen going from results for c.4.γ to c.5.γ. Ductility is relatively unchanged by the addition of graphite, or for porosity of 4%. Ductility increases from 0.047 to 0.052 for the larger porosity of 8%. Energy density drops consistently with peak stress for cases c.β.γ when β≤5: the rate of decrease of ${\bar{w}}_{C}$ with increasing defect fraction is more severe for pores than for graphite. Orientation-induced strength variations $\Delta {\bar{P}}_{C}$ are not notably different among any of the simulation sets in Table 7.

Cases c.6.γ are weakest in terms of strength (10.46 GPa), which might be anticipated given the superposition of porosity and graphitic inclusions in the corresponding microstructures. Less expected is the increase in strain at peak load ${\bar{\epsilon }}_{C}$, to 0.054, relative to the average of the baseline cases at 0.047. The increased compliance of the porous material, softened further by graphite, enables greater strain accommodation prior to attainment of peak load at which fractures percolate across the sample. This leads to a delay in fracture and a higher ${\bar{w}}_{C}$ relative to cases c.5.γ. Now compare results for cases c.3.γ of Table 6 containing 8% graphite distributed throughout SiC matrix and GB layer regions, with cases b.4.γ of Table 4 containing 11.6% graphite isolated in GB layer regions encasing larger diamond crystals. Peak stresses ${\bar{P}}_{C}$ of the latter are 13.69 GPa, energy ${\bar{w}}_{C}$ is 0.391 GJ/m${}^{3}$, and ductility ${\bar{\epsilon }}_{C}$ is 0.047. Peak values of the former (8% graphite, distributed) are 12.64 GPa, 0.358 GJ/m${}^{3}$, and 0.046. Thus, the distributed graphite is more deleterious to strength, toughness, and ductility than a higher concentration of graphite isolated only to local regions encasing diamond grains. In the more damaging case, the distributed graphite weakens the overall structure sufficiently to enable crack extension across the aggregate, leading to catastrophic failure at a lower applied strain. In the case with isolated graphite, fractures initiate where graphite exists at GB layers (e.g., see Figure 5), but crack extension is restricted by surrounding regions of SiC matrix, diamond, and SiC grains that are inherently stronger than graphite-infused material.

Summarizing, the present results show a decrease in peak compressive strength of around 7% with 8% graphite, a decrease in strength of 21% with 8% porosity, and a decrease of 23% with 5% graphite and 8% porosity. Such information can be used to inform materials synthesis, for example, fabrication controls to minimize graphite content while maintaining full density such that strength is maximized. Whether such efforts are pragmatic depends on manufacturing capabilities and cost effectiveness; these in turn depend on scale of production and other factors established by the industry and economy rather than the scientific community.

## 9. Conclusions

A phase field theory has been informed by characterization experiments, MD simulations, and micromechanics-based homogenization. The framework has been implemented to study deformation and failure of ceramic composites. The material consists of diamond and SiC, with a range of grain sizes and initial defects in the form of graphitic layers, graphite inclusions, and voids. Distributions of fracture energies for SiC-SiC and diamond-SiC interfaces have been obtained from MD simulations, where grain boundary character has been deduced from EBSD measurements on a representative material sample. Similarly, statistical grain size data have been used to construct a realistic FE rendering for phase field simulations. Simulation data from MD and experimental data from EBSD are new features not considered in prior phase field studies [23,24,25]. Baseline simulations have considered the most physically realistic microstructure, containing 70% diamond and 30% SiC, where the latter is distributed among discretely resolved grains, isotropic matrix, and grain boundary layers. Parametric studies have probed different microstructures containing different phase content, different fracture energy distributions, and different defect content (graphite and porosity). Notable findings are summarized below:

- Mean fracture energies of diamond-SiC interfaces predicted from MD simulations are substantially lower than those of SiC-SiC interfaces;
- Peak strength and tangent compressive stiffness of the composite increases with increasing diamond fraction when other physical properties are assumed fixed;
- In the absence of porosity or graphite, diamond-SiC boundaries are the weak link in the microstructure; strength and ductility are reduced when the relative fraction of diamond-SiC to SiC-SiC potential fracture sites increases;
- Peak strength and ductility of the composite are substantially reduced when graphitic layers encase large diamond crystals, and these properties are even more compromised when the same fraction of graphite is distributed throughout the whole microstructure;
- Low concentrations of pores and graphite have moderately deleterious effects on strength, but neither significantly reduces the strain at peak load, i.e., these defects do not substantially reduce ductility;
- High global concentrations of pores (8%) cause a much more drastic drop in strength and stiffness than the same global fraction of graphite;
- Twinning occurs in favorably oriented SiC crystals and is sensitive to lattice orientation distribution, but it does not notably alter the overall stress–strain response for the microstructures considered herein.

The above conclusions imply that diamond volume fraction should be maximized when processing diamond-SiC composites for exceptional compressive strength and stiffness, so long as the presence of diamond-SiC boundaries, graphite, and voids can be minimized, or at least not increased, with increasing diamond fraction. Graphite is less harmful to peak compressive strength than voids of the same volume fraction. Low porosity may be tolerable, but void fractions in excess of a threshold on the order of 5% are predicted to much more severely degrade overall strength and stiffness.

The present results on compressive behavior of diamond-SiC composites await quantitative validation. However, flexure strength and fracture toughness of CVI (chemical vapor infiltration) diamond-SiC both show an increase with increasing diamond fraction [13]. These findings are in qualitative agreement with the present simulation results that show increases in stiffness and compressive strength with increasing diamond fraction. Observed GB fractures in the SiC matrix and transgranular fractures in diamond [13] are also consistent with the present simulations. Data relating unconfined compressive strength to graphite and porosity in diamond-SiC are likewise not available from experiments. Indentation experiments [16] on fully dense diamond-SiC with graphitic layers show a hardness reduction as the graphite fraction increases from 0% to 0.4% to 5.3%. This decrease in hardness is qualitatively consistent with a decrease in compressive strength predicted by the present simulations. In three-point bending experiments [12], a decreasing flexure strength due to increasing pore size was proposed. Unconfined compressive strengths of pure SiC polycrystals predicted by the phase field approach (≈7 GPa) [23] are bounded by experimental data from macroscopic compression (5–6 GPa) [69,70] and micropillar compression (10 GPa) [71], providing confidence in the modeling techniques.

New experimentation on diamond-SiC material samples of similar size and composition is therefore required for precise validation. Conventional methods (e.g., compression, flexure) may be used for average behaviors, but novel techniques are required to validate local stress magnitudes that are predicted to exceed 40 GPa concentrated in local regions of microstructure. The extreme hardness and stiffness of diamond pose challenges for mechanical experiments, especially machining of samples.

Methods developed in this work, as well as general trends in results, may be applied towards comparative studies of other dual-phase ceramic systems, especially those in which one phase is notably harder or stiffer than another. In the present case, diamond is the harder and stiffer phase, with SiC the more compliant material. Other dual-phase ceramics of this category include those with boron carbide (B${}_{4}$C) serving as the harder, and often less ductile phase, and either SiC [106] or titanium diboride (TiB${}_{2}$) [107,108] the more ductile phase. Competing mechanisms of transgranular fracture in the harder phase, intergranular fracture, and fracture at phase boundaries, as well as possible partial dislocation slip [63,69,109] or deformation twinning [30,110], are likewise observed in these material systems.

Results reported herein should be useful for design and optimization of ultra-hard ceramics for mechanical applications. Notably, diamond- and boron-based ceramics are found in industrial applications requiring very high hardness and wear resistance [14,16], including aerospace and machining operations [15]. The diamond phase has the highest hardness and highest thermal conductivity of known natural materials [15]. The second phase (SiC in this case) increases fracture toughness, providing dynamic impact resistance [10]. Because of their unique thermal and mechanical properties, diamond-SiC composites are also of interest for high performance electronics, heat transport elements, tribological uses, and other structural systems where light weight and high stiffness are sought [15].

## Figures and Tables

**Table 1:** Physical properties or model parameters for cubic diamond (C), silicon carbide (β-SiC), and graphite (C). Values for isotropic matrix in parentheses. References in text of Section 3.1.

Parameter [Units]	Definition	Diamond	Silicon Carbide	Graphite
$\mu_{0}$ [GPa]	initial shear modulus	535	191	126
$\lambda_{0}$ [GPa]	initial Lamé modulus	85	97	76
Υ [J/m${}^{2}$]	fracture energy	5.30	2.33	0.20
Γ [J/m${}^{2}$]	twin boundary energy	-	0.034	-
$\hat{\beta }$	fracture anisotropy	100 (0)	100 (0)	0
$l/l_{G}$	regularization length / grain size	0.04	0.04	0.04
$\gamma_{0}$	max twinning shear	-	$\sqrt{2}/2$ (0)	-
$x_{\xi }$	max dilatation of bulking or cavitation	0 (0.04)	0 (0.04)	0
α,χ	kinematic interpolation	3,-	3,3	3,-

**Table 2:** Phase field simulation set α.β.γ, where “α” = a corresponds to cases of Section 3 and Section 4, “β” to a material distribution within a microstructure, and “γ” to loading direction *X*, *Y*, or *Z*.

Simulation Label α.β.γ	Diamond Grains num, vol %	SiC Grains num, vol %	SiC Matrix num, vol %	GB Layer num, vol %	GB Layer Material
a.1.x,y,z	701, 70.0%	178, 9.4%	177, 9.0%	170, 11.6%	SiC matrix
a.2.x,y,z	701, 70.0%	178, 9.4%	177, 9.0%	170, 11.6%	graphite
a.3.x,y,z	701, 70.0%	178, 9.4%	177, 9.0%	170, 11.6%	diamond matrix
a.4.x,y,z	701, 70.0%	355, 18.4%	0, 0.0%	170, 11.6%	SiC matrix
a.5.x,y,z	701, 70.0%	0, 0.0%	355, 18.4%	170, 11.6%	SiC matrix
a.6.x,y,z	170, 39.4%	355, 49.0%	0, 0.0%	170, 11.6%	SiC matrix

**Table 3:** Effects of phase content on peak average compressive stress ${\bar{P}}_{C}$ and corresponding strain ${\bar{\epsilon }}_{C}$ and stress work ${\bar{w}}_{C}$. Each row contains mean from three associated simulations a.β.x, a.β.y, and a.β.z, where β=1,…,6 (Table 2). Variation $\Delta {\bar{P}}_{C}$ is difference between min and max values among each set of three simulations x,y,z.

Simulation Set	${\bar{P}}_{C}$ [GPa]	${\bar{\epsilon }}_{C}$	${\bar{w}}_{C}$ [GJ/m${}^{3}$]	$\Delta {\bar{P}}_{C}$ [GPa]
a.1.x,y,z	16.91	0.052	0.517	0.39
a.2.x,y,z	14.44	0.047	0.399	0.49
a.3.x,y,z	19.00	0.052	0.580	0.23
a.4.x,y,z	16.85	0.051	0.502	0.28
a.5.x,y,z	16.79	0.049	0.465	0.76
a.6.x,y,z	12.07	0.048	0.341	0.62

**Table 4:** Phase field simulation set α.β.γ, where “α” = b corresponds to cases of Section 6, “β” to a material distribution within a microstructure, and “γ” to loading direction *X*, *Y*, or *Z*.

Simulation Label α.β.γ	Diamond Grains num, vol %	SiC Grains num, vol %	SiC Matrix ^a^ num, vol %	GB Layer ^b^ Material	Lattice Distrib.	MD Energy Distrib.
b.1.x,y,z	701, 70.0%	178, 9.4%	177, 9.0%	SiC matrix	I,II,III	1,2,3
b.2.x,y,z	701, 70.0%	178, 9.4%	177, 9.0%	SiC matrix	I,II,III	4,5,6
b.3.x,y,z	701, 70.0%	178, 9.4%	177, 9.0%	graphite	IV,V,VI	7,8,9
b.4.x,y,z	701, 70.0%	178, 9.4%	177, 9.0%	graphite	IV,V,VI	10,11,12
b.5.x,y,z	701, 70.0%	0, 0.0%	355, 18.4%	SiC matrix	VII,VIII,IX	13,14,15
b.6.x,y,z	701, 70.0%	0, 0.0%	355, 18.4%	SiC matrix	VII,VIII,IX	16,17,18

^a^ SiC-SiC GB fracture assumed for simulations b.1.*γ*, b.3.*γ*, b.5.*γ*; diamond-SiC fracture assumed for simulations b.2.*γ*, b.4.*γ*, b.6.*γ*, ^b^ diamond-SiC fracture if layer material is SiC matrix, otherwise graphite fracture.

**Table 5:** Effects of GB strength distributions on peak average compressive stress ${\bar{P}}_{C}$ and corresponding strain ${\bar{\epsilon }}_{C}$ and stress work ${\bar{w}}_{C}$. Each row contains mean from three associated simulations b.β.x, b.β.y, and b.β.z, where β=1,…,6 (Table 4). Variation $\Delta {\bar{P}}_{C}$ is difference between min and max values among each set of three simulations x,y,z.

Simulation Set	${\bar{P}}_{C}$ [GPa]	${\bar{\epsilon }}_{C}$	${\bar{w}}_{C}$ [GJ/m${}^{3}$]	$\Delta {\bar{P}}_{C}$ [GPa]
b.1.x,y,z	16.06	0.049	0.464	0.50
b.2.x,y,z	15.46	0.048	0.442	0.46
b.3.x,y,z	14.38	0.049	0.427	0.46
b.4.x,y,z	13.69	0.047	0.391	0.67
b.5.x,y,z	15.65	0.048	0.440	0.39
b.6.x,y,z	14.29	0.044	0.370	0.49

**Table 6:** Phase field simulation set α.β.γ, where “α” = c corresponds to cases of Section 8, “β” to a material distribution within a microstructure, and “γ” to loading direction *X*, *Y*, or *Z*. All simulations here include 701 diamond grains (70% by volume), 178 SiC grains (9.4% by volume), 177 SiC matrix regions (9.0% by volume), and 170 GB layer regions (11.6% by volume, including SiC with possible porosity and/or graphite).

Simulation Label α.β.γ	GB Layer ${}^{a}$ Material	Local Graphite $\upsilon_{g}$, Global vol %	Local Porosity φ, Global vol %
c.1.x,y,z	SiC	0, 0%	0, 0%
c.2.x,y,z	SiC + graphite	0.2, 4%	0, 0%
c.3.x,y,z	SiC + graphite	0.4, 8%	0, 0%
c.4.x,y,z	SiC + pores	0, 0%	0.2, 4%
c.5.x,y,z	SiC + pores	0, 0%	0.4, 8%
c.6.x,y,z	SiC + graphite + pores	0.4, 5%	0.4, 8%

^a^ Diamond-SiC strength from MD distributions, degraded by *φ* and/or *v_g_*.

**Table 7:** Effects of initial porosity and graphite inclusions on peak average compressive stress ${\bar{P}}_{C}$ and corresponding strain ${\bar{\epsilon }}_{C}$ and stress work ${\bar{w}}_{C}$. Each row contains mean from three associated simulations c.β.x, c.β.y, and c.β.z, where β=1,…,6 (Table 6). Variation $\Delta {\bar{P}}_{C}$ is difference between min and max values among each set of three simulations x,y,z.

Simulation Set	${\bar{P}}_{C}$ [GPa]	${\bar{\epsilon }}_{C}$	${\bar{w}}_{C}$ [GJ/m${}^{3}$]	$\Delta {\bar{P}}_{C}$ [GPa]
c.1.x,y,z	13.58	0.047	0.396	0.45
c.2.x,y,z	12.92	0.046	0.368	0.59
c.3.x,y,z	12.64	0.046	0.358	0.52
c.4.x,y,z	12.45	0.047	0.356	0.58
c.5.x,y,z	10.79	0.052	0.340	0.50
c.6.x,y,z	10.46	0.054	0.344	0.47

## Data Availability

Data will be provided by the authors upon request.
